# Mitigating co-circulation of seasonal influenza and COVID-19 pandemic in the presence of vaccination: A mathematical modeling approach

**DOI:** 10.3389/fpubh.2022.1086849

**Published:** 2023-01-04

**Authors:** Bushra Majeed, Jummy Funke David, Nicola Luigi Bragazzi, Zack McCarthy, Martin David Grunnill, Jane Heffernan, Jianhong Wu, Woldegebriel Assefa Woldegerima

**Affiliations:** ^1^Laboratory for Industrial and Applied Mathematics, Department of Mathematics and Statistics, York University, Toronto, ON, Canada; ^2^Centre for Disease Modeling, Department of Mathematics and Statistics, York University, Toronto, ON, Canada; ^3^Modelling Infection and Immunity Lab, Department of Mathematics and Statistics, York University, Toronto, ON, Canada

**Keywords:** COVID-19, influenza, co-circulation, seasonal flu, vaccine coverage, mathematical model

## Abstract

The co-circulation of two respiratory infections with similar symptoms in a population can significantly overburden a healthcare system by slowing the testing and treatment. The persistent emergence of contagious variants of SARS-CoV-2, along with imperfect vaccines and their waning protections, have increased the likelihood of new COVID-19 outbreaks taking place during a typical flu season. Here, we developed a mathematical model for the co-circulation dynamics of COVID-19 and influenza, under different scenarios of influenza vaccine coverage, COVID-19 vaccine booster coverage and efficacy, and testing capacity. We investigated the required minimal and optimal coverage of COVID-19 booster (third) and fourth doses, in conjunction with the influenza vaccine, to avoid the coincidence of infection peaks for both diseases in a single season. We show that the testing delay brought on by the high number of influenza cases impacts the dynamics of influenza and COVID-19 transmission. The earlier the peak of the flu season and the greater the number of infections with flu-like symptoms, the greater the risk of flu transmission, which slows down COVID-19 testing, resulting in the delay of complete isolation of patients with COVID-19 who have not been isolated before the clinical presentation of symptoms and have been continuing their normal daily activities. Furthermore, our simulations stress the importance of vaccine uptake for preventing infection, severe illness, and hospitalization at the individual level and for disease outbreak control at the population level to avoid putting strain on already weak and overwhelmed healthcare systems. As such, ensuring optimal vaccine coverage for COVID-19 and influenza to reduce the burden of these infections is paramount. We showed that by keeping the influenza vaccine coverage about 35% and increasing the coverage of booster or fourth dose of COVID-19 not only reduces the infections with COVID-19 but also can delay its peak time. If the influenza vaccine coverage is increased to 55%, unexpectedly, it increases the peak size of influenza infections slightly, while it reduces the peak size of COVID-19 as well as significantly delays the peaks of both of these diseases. Mask-wearing coupled with a moderate increase in the vaccine uptake may mitigate COVID-19 and prevent an influenza outbreak.

## 1. Introduction

Despite the implementation of non-pharmaceutical interventions (NPIs) (1, 2) and the existence of highly effective vaccines (3), the “coronavirus disease 2019” (COVID-19) pandemic continues to plague the globe (4). Due to the emergence of multiple highly contagious strains (5) that can evade the immune response and make the existing vaccines less effective (6, 7), it can be expected that new waves of COVID-19 will arise (8), with COVID-19 becoming an endemic disease (9). If these waves occur during a typical influenza season in many regions of the world, then this would create a situation of co-circulation of multiple respiratory viruses, including influenza and respiratory syncytial virus (RSV) (10), among others. Since respiratory pathogens share similar symptoms, this poses a serious challenge to the global public health system (11). During the first 2 years of the still ongoing COVID-19 pandemic, seasonal influenza infections have been mitigated, likely due to the mandatory use of personal protective equipment (PPE) and the implementation of stringent packages of NPIs to contain the spread of COVID-19 (12). On the other hand, the lack of exposure to the influenza virus may also have decreased the population's immunity levels and increased susceptibility to influenza because of its low circulation in the two previous seasons (13). All this, taken together, may potentially lead to a larger seasonal influenza outbreak when COVID-19-induced social distancing and other restrictions are relaxed, creating the ideal situation for influenza–COVID co-circulating in the population.

The combined risk of the concurrent influenza epidemic and the COVID-19 pandemic is a serious global public health concern since it can be extremely difficult to anticipate influenza circulation in the upcoming winter with COVID-19. Some epidemiological observational studies have investigated the impact of SARS-CoV-2 and influenza viruses co-circulation in terms of prevalence rate of co-circulation, clinical outcomes, and imposed burden (11, 14).

Mathematical modeling can play a key role in accounting for interactions of a given pathogen with other infectious agents and in quantifying the real burden of each pathogen and the full impact and effectiveness of public health interventions targeting each infectious agent (15, 16). This is particularly relevant given that, in the current situation, where the SARS-CoV-2 virus is still transmitting continuously in the world despite the availability of many effective vaccines, the emergence of new variants of concern (VOCs) is inevitable.

Considering the additional burden of COVID-19 during the influenza season on population health and healthcare systems, including emergency departments (EDs), it is of paramount importance to investigate the effects of co-circulations when vaccines are available for both diseases. Therefore, the present mathematical model was developed with the aim of studying the impact of SARS-CoV-2 during an influenza season, quantitatively assessing the effects of the co-circulation of the two respiratory pathogens. In particular, in the present study, we are interested in finding optimal strategies to manage and control both influenza and COVID-19 outbreaks during the same season. Among many scenarios and interventions, we consider optimal strategies to delay and separate the peaks of the influenza outbreak and COVID-19 wave.

## 2. The co-circulation model

To investigate the co-circulation of influenza and SARS-CoV-2, a deterministic compartmental model formulated in terms of ordinary differential equations was employed. The objective of the study is to identify potential control strategies to mitigate the burden caused by both viruses in the near term, that is, during a single respiratory illness season, several simplifying assumptions were made to focus on essential elements relevant to the present study. Specifically, the following assumptions were made to enable such a focus. Changes in population demographics were not considered, that is, births and deaths were not modeled, and the population size remained constant. A closed population was considered and, therefore, no inbound or outbound travel occurred. It was assumed that the population mixed homogeneously. Age-related heterogeneities (e.g., susceptibility to infection, social contact mixing, and vaccination coverage) and spatial heterogeneities (e.g., testing and case reporting, social contact mixing, and level of pathogen circulation) were not considered. Given that symptomatic influenza and COVID-19 share similar symptoms, the RT-PCR testing capacity was considered to be a shared resource between infections occurring by both influenza and SARS-CoV-2.

Due to the scarcity of data and for model simplification, we assume that infections are exclusive and neither pathogen can be supplanted within an infected host. In other words, we assume no co-infection and super-infection of both diseases. However, secondary infection is possible, that is, an individual, after recovery from one disease, can get infected by other diseases. Focusing on a single respiratory illness reason, it was assumed that upon infection by both influenza virus and SARS-CoV-2 (temporally distinct infections) complete immunity against infection by both pathogens was conferred. All individuals were assumed to be vaccinated before the considered influenza season started, and no vaccination occurred during the season. This means that, rather than modeling vaccination as a time-dependent process, administration of vaccines was modeled as a time-independent process and was embedded in the initial conditions.

**Figure 1:**
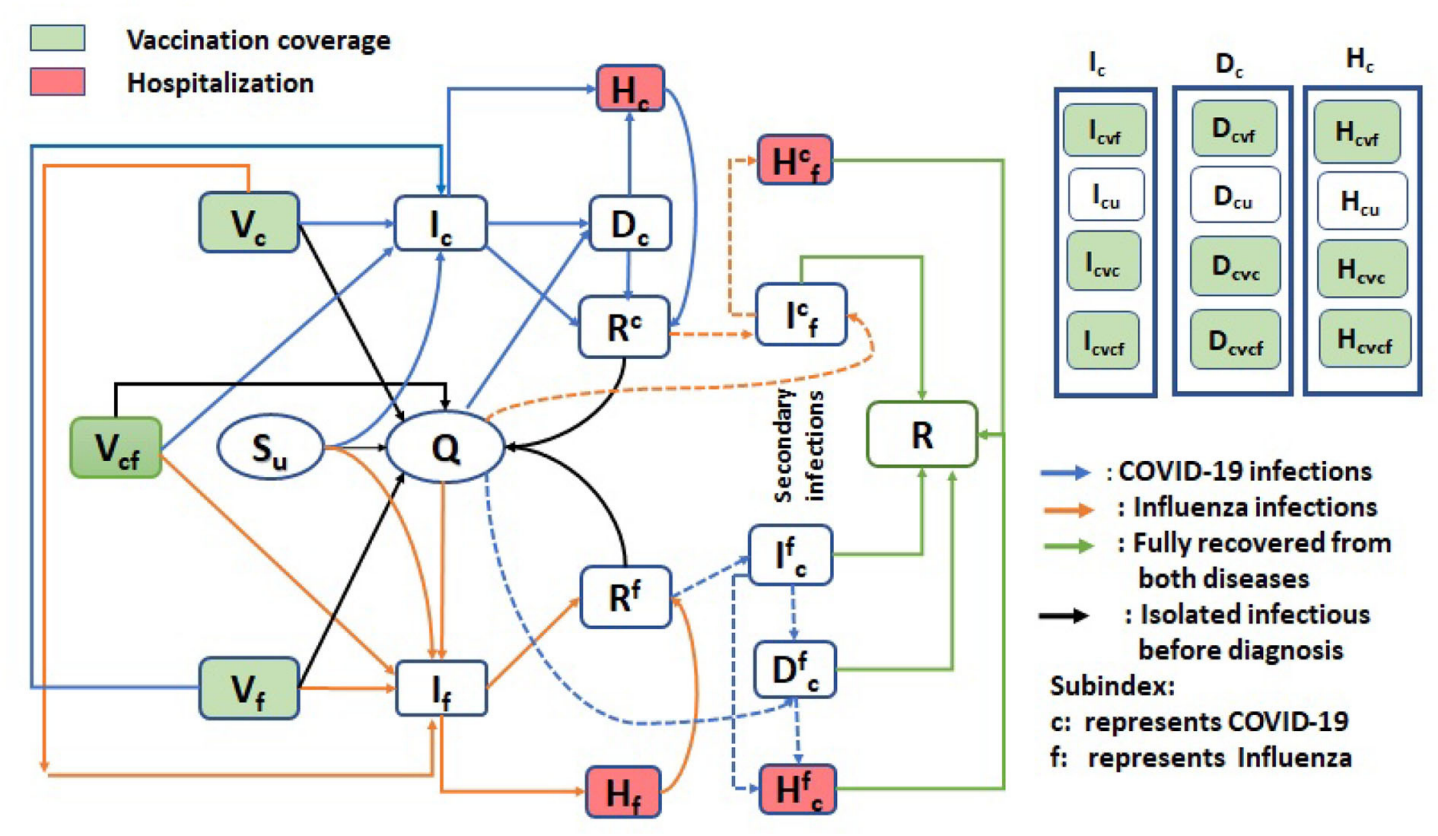
A flow diagram of the transmission dynamics model, considering the co-circulation of influenza and SARS-CoV-2 viruses. On the right side of the diagram, classes *I*_*c*_, *D*_*c*_, and *H*_*c*_ represent that there are further sub-classes in each of these compartments. Similarly, flu classes have sub classes.

The population was stratified into susceptible, isolated, infected, diagnosed, recovered, and hospitalized states, with further stratification based on epidemiological history (e.g., prior infection) of the two circulating viruses (Figure 1). Vaccination of individuals against both influenza and COVID-19 was considered in the model; therefore, dividing the population susceptible to infection by both pathogens into four classes: those individuals not vaccinated against either influenza or COVID-19 (denoted by *S*_*u*_); those individuals vaccinated against COVID-19 and not influenza (*V*_*c*_); those individuals vaccinated against influenza and not COVID-19 (*V*_*f*_); and those individuals vaccinated against influenza and COVID-19 (*V*_*cf*_). Each of these four groups could potentially be infected by SARS-CoV-2 and influenza, with vaccination modulating the infection risk in each class.

In the model, unvaccinated individuals (*S*_*u*_) were infected by influenza at rate λ_*f*_ and with SARS-CoV-2 at rate λ_*c*_. Vaccination status modulated the infection process as follows: Individuals vaccinated against influenza were assumed to be infected by influenza at a reduced rate (1−ϕ_*f*_)λ_*f*_, where ϕ_*f*_ is the vaccine effectiveness against infection by influenza. It has been suggested that influenza vaccination may reduce susceptibility to COVID-19 (17, 18); therefore, those individuals vaccinated against influenza were assumed to be infected by SARS-CoV-2 at the reduced rate (1−η)λ_*c*_, where 0 ≤ η ≤ 1. Individuals vaccinated against both COVID-19 and influenza were therefore infected by SARS-CoV-2 at rate λ_*c*_(1−ϕ_*c*_)(1−η) and by influenza virus at rate (1−ϕ_*f*_)λ_*f*_.

Upon infection, and in light of the fact that influenza and COVID-19 share common symptoms, it was assumed that a fraction *q* of symptomatic individuals isolate themselves until their test results become available (thus entering the Q class); meanwhile, the remaining proportion (1−*q*) continues socio-economic activity without disruption (i.e., their contact patterns were assumed to remain unaltered). Those individuals in the isolated (*Q*) class that tested positive for COVID-19 were assumed to remain isolated and not infectious; meanwhile, those individuals who tested negative were assumed to end their isolation and resume normal mixing.

Those individuals in *I*_*c*_ are diagnosed and isolated, recovered naturally, or hospitalized from their primary infection. This is in contrast to individuals in *I*_*f*_ who either recover naturally or are hospitalized from their primary infection. Given that the study is concerned with a single respiratory disease season, we considered the case where recovery from this primary infection yields immunity to re-infection by the same pathogen. Meanwhile, those who recovered from primary infection (*R*^*f*^ and *R*^*c*^) become susceptible to secondary infection. When an individual experiences primary and secondary infection, they are considered immune to infection by both influenza and SARS-CoV-2.

Infectious individuals, either influenza (*I*_*f*_) or COVID-19 (*I*_*c*_), may be diagnosed, and subsequently, COVID-19-tested positive individuals are isolated (moving to (*D*_*c*_) compartment), while COVID-19-tested negative will move out from the *Q* class to (*I*_*f*_) compartment. Later on, we will specify how the incidence of both diseases impacts the diagnosis speed since they share common symptoms.

Isolated individuals were assumed to transmit the disease longer. Furthermore, diagnosed (*D*_*c*_) and infectious individuals (*I*_*f*_) or (*I*_*c*_) can recover from influenza or COVID-19, respectively, at the rate of γ_*cD*_ and γ_*fI*_ or γ_*cI*_ (moving to class *R*^*f*^ or *R*^*c*^, respectively), or they will be admitted to hospitals with disease-induced severity, at the rate of τ_*f*_ from *I*_*f*_ class and at the rate of θ_*c*_ and τ_*c*_ from *D*_*c*_ and *I*_*c*_ classes, respectively.

We assume that after recovery from either of these diseases, an individual is completely immune (recall this research focuses on a flu season only) to that disease but susceptible to the other diseases. Individuals recovered from COVID-19 but infected with influenza are denoted by $I_{f}^{c}$ and hospitalized as $H_{f}^{c}$. And those individuals recovered from influenza but infected with COVID-19 are denoted by $I_{c}^{f}$, with the diagnosed and isolated denoted by $D_{c}^{f}$ and hospitalized $H_{c}^{f}$. Individuals experiencing such secondary infections become immune to both diseases after recovery from the second disease.

### 2.1. Standing assumptions

We assume that a fraction of the population receives the flu vaccine, and another fraction of the population receives the COVID-19 vaccine. A fraction of the population may receive vaccines for both influenza and COVID-19. We assume that they receive the vaccine(s) before our considered influenza season starts. We do not assume a perfect vaccine, so individuals who received a vaccine can still be infected by the disease intended. Furthermore, we assume that a vaccine against influenza or COVID-19 reduces the risk of being infected (reduction in susceptibility) and reduces the risk of severity of disease (hospitalization) if infected by the disease the vaccine is intended.

The flow diagram of the model formulation is shown in Figure 1. Note that in each class other than susceptibles (*S*_*u*_, *V*_*c*_, *V*_*f*_, and *V*_*cf*_) and completely recovered *R* classes, there are further four sub-classes that are tracked. For instance, the *I*_*c*_ class has *I*_*cvc*_, *I*_*cvf*_, *I*_*cu*_, and *I*_*cvcf*_ sub-classes. In these sub-classes, the first letter in sub-index represents the disease class “f” for flu and “c” for COVID-19. Other indices represent the status of the vaccine. For example,

*I*_*cvc*_: First letter “c” in sub-index means infectious with COVID-19, next “*vc*” represents vaccinated against COVID-19.
*I*_*cvf*_: First letter “c” in sub-index means infectious with COVID-19, next “*vf*” represents vaccinated against flu.
*I*_*cu*_: First letter “c” in sub-index means infectious with COVID-19, next “u” represents unvaccinated.
*I*_*cvcf*_: First letter “c” in sub-index means infectious with COVID-19, next “*vcf*” represents vaccinated against both flu and COVID.

Due to the co-circulation of both influenza and SARS-CoV-2 in a single season and due to their common symptoms, COVID-19 testing can be slowed down with a large number of individuals infected with either flu and COVID-19. Therefore, the diagnostic rate to confirm the disease type is a decreasing function of the total number of infections (with either flu or COVID-19) modeled by the available testing capacity. This can be modeled using the Holling type II functions:


$$\begin{array}{l} F_{c}\left(I_{c},I_{f},I_{f}^{c},I_{c}^{f},Q\right)=\frac{\delta_{Ic}I_{c}}{1+w\left(I_{c}+I_{f}+I_{f}^{c}+I_{c}^{f}+Q\right)}\\ F_{f}\left(I_{c},I_{f},I_{f}^{c},I_{c}^{f},Q\right)=\frac{\delta_{If}I_{f}}{1+w\left(I_{c}+I_{f}+I_{f}^{c}+I_{c}^{f}+Q\right)}\\ F_{f}^{c}\left(I_{c},I_{f},I_{f}^{c},I_{c}^{f},Q\right)=\frac{\delta_{If}I_{f}^{c}}{1+w\left(I_{c}+I_{f}+I_{f}^{c}+I_{c}^{f}+Q\right)}\\ F_{c}^{f}\left(I_{c},I_{f},I_{f}^{c},I_{c}^{f},Q\right)=\frac{\delta_{Ic}I_{c}^{f}}{1+w\left(I_{c}+I_{f}+I_{f}^{c}+I_{c}^{f}+Q\right)}\\ F_{Q}\left(I_{c},I_{f},I_{f}^{c},I_{c}^{f},Q\right)=\frac{\delta_{Q}Q}{1+w\left(I_{c}+I_{f}+I_{f}^{c}+I_{c}^{f}+Q\right)}, \end{array}$$

where *w* is the constant such that 1/*w* is the maximum number of people who can be tested per day (testing capacity per day). These functions characterize the saturation phenomenon of limited testing resources.

#### 2.1.1. Forces of infections: COVID-19 and influenza

We use standard incidence to describe the forces of infections:


$$\begin{array}{l} \lambda_{c}=\frac{\beta_{c}C\left(I_{cu}+I_{cvf}+I_{cvc}+I_{cvcf}+I_{cu}^{f}+I_{cvf}^{f}+I_{cvc}^{f}+I_{cvcf}^{f}\right)}{N} \end{array}$$

$$\begin{array}{l} \lambda_{f}=\frac{\beta_{f}C\left(I_{fu}+I_{fvf}+I_{fvc}+I_{fvcf}+I_{fu}^{c}+I_{fvf}^{c}+I_{fvc}^{c}+I_{fvcf}^{c}\right)}{N}. \end{array}$$

In summary, the mathematical model for the co-circulation of COVID-19 and flu is given below, with two diseases coupled through their impact on testing speed (and thus isolation duration of the patients with flu):


(1) $$\begin{array}{l} S_{u}^{\prime }=-\lambda_{c}S_{u}-\lambda_{f}S_{u} \end{array}$$

(2) $$\begin{array}{l} V_{c}^{\prime }=-\lambda_{c}\left(1-\phi_{c}\right)V_{c}-\lambda_{f}V_{c} \end{array}$$

(3) $$\begin{array}{l} V_{f}^{\prime }=-\lambda_{f}\left(1-\phi_{f}\right)V_{f}-\lambda_{c}\left(1-\eta \right)V_{f} \end{array}$$

(4) $$\begin{array}{l} V_{cf}^{\prime }=-\lambda_{f}\left(1-\phi_{f}\right)V_{cf}-\lambda_{c}\left(1-\phi_{c}\right)\left(1-\eta \right)V_{cf} \end{array}$$

Note that there are 16 subclasses in Q compartment, that is,


$$\begin{array}{l} \begin{array}{r} Q^{\prime }=Q_{cvc}^{\prime }+Q_{cvf}^{\prime }+Q_{cu}^{\prime }+Q_{cvcf}^{\prime }+Q_{fvc}^{\prime }+Q_{fvf}^{\prime }+Q_{fu}^{\prime }+Q_{fvcf}^{\prime }+\\ Q_{fvc}^{\prime c}+Q_{fu}^{\prime c}+Q_{fvf}^{\prime c}+Q_{fvcf}^{\prime c}+Q_{cvc}^{\prime f}+Q_{cu}^{\prime f}+Q_{cvf}^{\prime f}+Q_{cvcf}^{\prime f} \end{array} \end{array}$$

where,


(5) $$\begin{array}{l} \{\begin{array}{l} \;Q_{cvc}^{\prime }=q\lambda_{c}\left(1-\phi_{c}\right)V_{c}-F_{Qcvc}\\ \;Q_{cvf}^{\prime }=q\lambda_{c}\left(1-\eta \right)V_{f}-F_{Qcvf}\\ \;Q_{cu}^{\prime }=q\lambda_{c}S_{u}-F_{Qcu}\\ Q_{cvcf}^{\prime }=q\lambda_{c}\left(1-\phi_{c}\right)\left(1-\eta \right)V_{cf}-F_{Qcvcf}\\ \;Q_{fvc}^{\prime }=q\lambda_{f}V_{f}-F_{Qfvc}\\ \;Q_{fvf}^{\prime }=q\lambda_{f}\left(1-\phi_{f}\right)V_{f}-F_{Qfvf}\\ \;Q_{fu}^{\prime }=q\lambda_{f}S_{f}-F_{Qfu}\\ Q_{fvcf}^{\prime }=q\lambda_{f}\left(1-\phi_{f}\right)V_{cf}-F_{Qfvcf}\\ \;Q_{fvc}^{\prime c}=q\lambda_{f}R_{vc}^{c}-F_{Qfvc}^{c}\\ \;Q_{fvf}^{\prime c}=q\lambda_{f}\left(1-\phi_{f}\right)R_{vf}^{c}-F_{Qfvf}^{c}\\ \;Q_{fu}^{\prime c}=q\lambda_{f}R_{u}^{c}-F_{Qfu}^{c}\\ Q_{fvcf}^{\prime c}=q\lambda_{f}\left(1-\phi_{f}\right)R_{vcf}^{c}-F_{fvcf}^{c}\\ \;Q_{cvc}^{\prime f}=q\lambda_{c}\left(1-\phi_{c}\right)R_{vc}^{f}-F_{Qcvc}^{f}\\ \;Q_{cvf}^{\prime f}=q\lambda_{c}\left(1-\eta \right)R_{vf}^{f}-F_{Qcvf}^{f}\\ \;Q_{cu}^{\prime f}=q\lambda_{c}R_{u}^{f}-F_{Qcu}^{f}\\ Q_{cvcf}^{\prime f}=q\lambda_{c}\left(1-\phi_{c}\right)\left(1-\eta \right)R_{vcf}^{f}-F_{Qcvcf}^{f} \end{array} \end{array}$$

Furthermore, we consider subclasses of *I*_*c*_ as follows:


$$\begin{array}{c} I_{c}^{\prime }=I_{cu}^{\prime }+I_{cvf}^{\prime }+I_{cvc}^{\prime }+I_{cvcf}^{\prime } \end{array}$$

(6) $$\begin{array}{l} \{\begin{array}{l} \;I_{cvc}^{\prime }=\left(1-q\right)\lambda_{c}\left(1-\phi_{c}\right)V_{c}-\gamma_{c}I_{cvc}-F_{cvc}\left(I_{c},I_{f},I_{f}^{c},I_{c}^{f},Q\right)\\ \;-\left(1-\kappa_{c}\right)\tau_{c}I_{cvc}\\ \;I_{cvf}^{\prime }=\left(1-q\right)\lambda_{c}\left(1-\eta \right)V_{f}-\gamma_{c}I_{cvf}-F_{cvf}\left(I_{c},I_{f},I_{f}^{c},I_{c}^{f},Q\right)\\ \;-\tau_{c}I_{cvf}\\ \;I_{cu}^{\prime }=\left(1-q\right)\lambda_{c}S_{u}-\gamma_{c}I_{cu}-F_{cu}\left(I_{c},I_{f},I_{f}^{c},I_{c}^{f},Q\right)-\tau_{c}I_{cu}\\ I_{cvcf}^{\prime }=\left(1-q\right)\lambda_{c}\left(1-\phi_{c}\right)\left(1-\eta \right)V_{cf}-\gamma_{c}I_{cvcf}\\ \;-F_{cvcf}\left(I_{c},I_{f},I_{f}^{c},I_{c}^{f},Q\right)-\left(1-\kappa_{c}\right)\tau_{c}I_{cvcf} \end{array} \end{array}$$

$$\begin{array}{c} D_{c}^{\prime }=D_{cu}^{\prime }+D_{cvf}^{\prime }+D_{cvc}^{\prime }+D_{cvcf}^{\prime } \end{array}$$

(7) $$\begin{array}{l} \{\begin{array}{l} \;D_{cvc}^{\prime }=F_{cvc}\left(I_{c},I_{f},I_{f}^{c},I_{c}^{f},Q\right)+F_{Qcvc}\left(I_{c},I_{f},I_{f}^{c},I_{c}^{f},Q\right)\\ \;-\gamma_{cD}D_{cvc}-\left(1-\kappa_{c}\right)\theta_{c}D_{cvc}\\ \;D_{cvf}^{\prime }=F_{cvf}\left(I_{c},I_{f},I_{f}^{c},I_{c}^{f},Q\right)+F_{Qcvf}\left(I_{c},I_{f},I_{f}^{c},I_{c}^{f},Q\right)\\ \;-\gamma_{cD}D_{cvf}-\theta_{c}D_{cvf}\\ \;D_{cu}^{\prime }=F_{cu}\left(I_{c},I_{f},I_{f}^{c},I_{c}^{f},Q\right)+F_{Qcu}\left(I_{c},I_{f},I_{f}^{c},I_{c}^{f},Q\right)\\ \;-\gamma_{cD}D_{cu}-\theta_{c}D_{cu}\\ D_{cvcf}^{\prime }=F_{cvcf}\left(I_{c},I_{f},I_{f}^{c},I_{c}^{f},Q\right)+F_{Qcvcf}\left(I_{c},I_{f},I_{f}^{c},I_{c}^{f},Q\right)\\ \;-\gamma_{cD}D_{cvcf}-\left(1-\kappa_{c}\right)\theta_{c}D_{cvcf} \end{array} \end{array}$$

$$\begin{array}{c} H_{c}^{\prime }=H_{cu}^{\prime }+H_{cvf}^{\prime }+H_{cvc}^{\prime }+H_{cvcf}^{\prime } \end{array}$$

(8) $$\begin{array}{l} \{\begin{array}{l} \;H_{cvc}^{\prime }=\left(1-\kappa_{c}\right)\theta_{c}D_{cvc}+\left(1-\kappa_{c}\right)\tau_{c}I_{cvc}-\gamma_{cH}H_{cvc}\\ \;H_{cvf}^{\prime }=\theta_{c}D_{cvf}+\tau_{c}I_{cvf}-\gamma_{cH}H_{cvf}\\ \;H_{cu}^{\prime }=\theta_{c}D_{cu}+\tau_{c}I_{cu}-\gamma_{cH}H_{cu}\\ H_{cvcf}^{\prime }=\left(1-\kappa_{c}\right)\theta_{c}D_{cvcf}+\left(1-\kappa_{c}\right)\tau_{c}I_{cvcf}-\gamma_{cH}H_{cvcf}. \end{array} \end{array}$$

Here, confirmed/diagnosed cases go to hospitals at rate θ_*c*_ and infected but not diagnosed at rate τ_*c*_, and κ_*c*_ is the effectiveness of the COVID vaccine to the severity of infection to breakthrough infection.


$$\begin{array}{c} R^{\prime c}=R_{vc}^{\prime c}+R_{u}^{\prime c}+R_{vf}^{\prime c}+R_{vcf}^{\prime c} \end{array}$$

(9) $$\begin{array}{l} \{\begin{array}{l} \;R_{vc}^{\prime c}=-\lambda_{f}R_{vc}^{c}+\gamma_{cH}H_{cvc}+\gamma_{cD}D_{cvc}+\gamma_{c}I_{cvc}\\ \;R_{vf}^{\prime c}=-\lambda_{f}\left(1-\phi_{f}\right)R_{vf}^{c}+\gamma_{cH}H_{cvf}+\gamma_{cD}D_{cvf}+\gamma_{c}I_{cvf}\\ \;R_{u}^{\prime c}=-\lambda_{f}R_{u}^{c}+\gamma_{cH}H_{cu}+\gamma_{cD}D_{cu}+\gamma_{c}I_{cu}\\ R_{vcf}^{\prime c}=-\lambda_{f}\left(1-\phi_{f}\right)R_{vcf}^{c}+\gamma_{cH}H_{cvcf}+\gamma_{cD}D_{cvf}+\gamma_{c}I_{cvcf} \end{array} \end{array}$$

$$\begin{array}{c} I_{f}^{\prime c}=I_{fvc}^{\prime c}+I_{fu}^{\prime c}+I_{fvf}^{\prime c}+I_{fvcf}^{\prime c} \end{array}$$

(10) $$\begin{array}{l} \{\begin{array}{l} \;I_{fvc}^{\prime c}=\left(1-q\right)\lambda_{f}R_{vc}^{c}-\gamma_{f}I_{fvc}^{c}-\tau_{f}I_{fvc}^{c}+F_{Qfvc}^{c}\left(I_{c},I_{f},I_{f}^{c},I_{c}^{f},Q\right)\\ \;I_{fvf}^{\prime c}=\left(1-q\right)\lambda_{f}\left(1-\phi_{f}\right)R_{vf}^{c}-\gamma_{f}I_{fvf}^{c}-\tau_{f}\left(1-\kappa_{f}\right)I_{fvf}^{c}\\ \;+F_{Qfvf}^{c}\left(I_{c},I_{f},I_{f}^{c},I_{c}^{f},Q\right)\\ \;I_{fu}^{\prime c}=\left(1-q\right)\lambda_{f}R_{u}^{c}-\gamma_{f}I_{fu}^{c}-\tau_{f}I_{fu}^{c}+F_{Qfu}^{c}\left(I_{c},I_{f},I_{f}^{c},I_{c}^{f},Q\right)\\ I_{fvcf}^{\prime c}=\left(1-q\right)\lambda_{f}\left(1-\phi_{f}\right)R_{vcf}^{c}-\gamma_{f}I_{fvcf}^{c}-\tau_{f}\left(1-\kappa_{f}\right)I_{fvcf}^{c}\\ \;+F_{fvcf}^{c}\left(I_{c},I_{f},I_{f}^{c},I_{c}^{f},Q\right) \end{array} \end{array}$$

$$\begin{array}{c} H_{f}^{\prime c}=H_{fvc}^{\prime c}+H_{fu}^{\prime c}+H_{fvf}^{\prime c}+H_{fvcf}^{\prime c} \end{array}$$

(11) $$\begin{array}{l} \{\begin{array}{l} \;H_{fvc}^{\prime c}=\tau_{f}I_{fvc}^{c}-\gamma_{fH}H_{fvc}^{c}\\ \;H_{fvf}^{\prime c}=\tau_{f}\left(1-\kappa_{f}\right)I_{fvf}^{c}-\gamma_{fH}H_{fvf}^{c}\\ \;H_{fu}^{\prime c}=\tau_{f}I_{fu}^{c}-\gamma_{fH}H_{fu}^{c}\\ H_{fvcf}^{\prime c}=\tau_{f}\left(1-\kappa_{f}\right)I_{fvcf}^{c}-\gamma_{fH}H_{fvcf}^{c} \end{array} \end{array}$$

$$\begin{array}{c} I_{f}^{\prime }=I_{fvc}^{\prime }+I_{fu}^{\prime }+I_{fvf}^{\prime }+I_{fvcf}^{\prime } \end{array}$$

(12) $$\begin{array}{l} \{\begin{array}{l} \;I_{fvc}^{\prime }=\left(1-q\right)\lambda_{f}V_{c}-\gamma_{f}I_{fvc}+F_{Qfvc}\left(I_{c},I_{f},I_{f}^{c},I_{c}^{f}\right)-\tau_{f}I_{fvc}\\ \;I_{fvf}^{\prime }=\left(1-q\right)\lambda_{f}\left(1-\phi_{f}\right)V_{f}-\gamma_{f}I_{fvf}+F_{Qfvf}\left(I_{c},I_{f},I_{f}^{c},I_{c}^{f}\right)\\ \;-\tau_{f}\left(1-\kappa_{f}\right)I_{fvf}\\ \;I_{fu}^{\prime }=\left(1-q\right)\lambda_{f}S_{u}-\gamma_{f}I_{fu}+F_{Qfu}\left(I_{c},I_{f},I_{f}^{c},I_{c}^{f}\right)-\tau_{f}I_{fu}\\ I_{fvcf}^{\prime }=\left(1-q\right)\lambda_{f}\left(1-\phi_{f}\right)V_{cf}-\gamma_{f}I_{cvcf}+F_{Qfvcf}\left(I_{c},I_{f},I_{f}^{c},I_{c}^{f}\right)\\ \;-\tau_{f}\left(1-\kappa_{f}\right)I_{cvcf} \end{array} \end{array}$$

$$\begin{array}{c} H_{f}^{\prime }=H_{fvc}^{\prime }+H_{fu}^{\prime }+H_{fvf}^{\prime }+H_{fvcf}^{\prime } \end{array}$$

(13) $$\begin{array}{l} \{\begin{array}{l} \;H_{fvc}^{\prime }=\tau_{f}I_{fvc}-\gamma_{fH}H_{fvc}\\ \;H_{fvf}^{\prime }=\tau_{f}\left(1-\kappa_{f}\right)I_{fvf}-\gamma_{fH}H_{fvf}\\ \;H_{fu}^{\prime }=\tau_{f}I_{fu}-\gamma_{fH}H_{fu}\\ H_{fvcf}^{\prime }=\tau_{f}\left(1-\kappa_{f}\right)I_{fvcf}-\gamma_{fH}H_{fvcf} \end{array} \end{array}$$

$$\begin{array}{c} R^{\prime f}=R_{vc}^{\prime f}+R_{u}^{\prime f}+R_{vf}^{\prime f}+R_{vcf}^{\prime f} \end{array}$$

(14) $$\begin{array}{l} \{\begin{array}{l} \;R_{vc}^{\prime f}=-\lambda_{c}\left(1-\phi_{c}\right)R_{vc}^{f}+\gamma_{fH}H_{fvc}+\gamma_{f}I_{fvc}\\ \;R_{vf}^{\prime f}=-\lambda_{c}\left(1-\eta \right)R_{vf}^{f}+\gamma_{fH}H_{fvf}+\gamma_{f}I_{fvf}\\ \;R_{u}^{\prime f}=-\lambda_{c}R_{u}^{f}+\gamma_{fH}H_{fu}+\gamma_{f}I_{fu}\\ R_{vcf}^{\prime f}=-\lambda_{c}\left(1-\phi_{c}\right)R_{vcf}^{f}+\gamma_{fD}D_{fvcf}+\gamma_{f}I_{fvcf} \end{array} \end{array}$$

$$\begin{array}{c} I_{c}^{\prime f}=I_{cvc}^{\prime f}+I_{cu}^{\prime f}+I_{cvf}^{\prime f}+I_{cvcf}^{\prime f} \end{array}$$

(15) $$\begin{array}{l} \{\begin{array}{l} \;I_{cvc}^{\prime f}=\left(1-q\right)\lambda_{c}\left(1-\phi_{c}\right)R_{vc}^{f}-\gamma_{c}I_{cvc}^{f}-\tau_{c}\left(1-\kappa_{c}\right)I_{cvc}^{f}\\ \;-F_{cvc}^{f}\left(I_{c},I_{f},I_{f}^{c},I_{c}^{f}\right)\\ \;I_{cvf}^{\prime f}=\left(1-q\right)\lambda_{c}\left(1-\eta \right)R_{vf}^{f}-\gamma_{c}I_{cvf}^{f}-\tau_{c}I_{cvf}^{f}\\ \;-F_{cvf}^{f}\left(I_{c},I_{f},I_{f}^{c},I_{c}^{f}\right)\\ \;I_{cu}^{\prime f}=\left(1-q\right)\lambda_{c}R_{u}^{f}-\gamma_{c}I_{cu}^{f}-\tau_{c}I_{cu}^{f}-F_{cu}^{f}\left(I_{c},I_{f},I_{f}^{c},I_{c}^{f}\right)\\ I_{cvcf}^{\prime f}=\left(1-q\right)\lambda_{c}\left(1-\phi_{c}\right)\left(1-\eta \right)R_{vcf}^{f}-\gamma_{c}I_{cvcf}^{f}-\tau_{c}\left(1-\kappa_{c}\right)\\ \;I_{cvcf}^{f}-F_{cvcf}^{f}\left(I_{c},I_{f},I_{f}^{c},I_{c}^{f}\right) \end{array} \end{array}$$

$$\begin{array}{c} D_{c}^{\prime f}=D_{cvc}^{\prime f}+D_{cu}^{\prime f}+D_{cvf}^{\prime f}+D_{cvcf}^{\prime f} \end{array}$$

(16) $$\begin{array}{l} \{\begin{array}{l} \;D_{cvc}^{\prime f}=F_{cvc}^{f}\left(I_{c},I_{f},I_{f}^{c},I_{c}^{f}\right)+F_{Qcvc}^{f}-\gamma_{cD}D_{cvc}^{f}-\theta_{c}\left(1-\kappa_{c}\right)D_{cvc}^{f}\\ \;D_{cvf}^{\prime f}=F_{cvf}^{f}\left(I_{c},I_{f},I_{f}^{c},I_{c}^{f}\right)+F_{Qcvf}^{f}-\gamma_{cD}D_{cvf}^{f}-\theta_{c}D_{cvf}^{f}\\ \;D_{cu}^{\prime f}=F_{cu}^{f}\left(I_{c},I_{f},I_{f}^{c},I_{c}^{f}\right)+F_{Qu}^{f}-\gamma_{cD}D_{fu}^{c}-\theta_{c}D_{cu}^{f}\\ D_{cvcf}^{\prime f}=F_{cvcf}^{f}\left(I_{c},I_{f},I_{f}^{c},I_{c}^{f}\right)+F_{Qcvcf}^{f}-\gamma_{cD}D_{fvcf}^{c}-\theta_{c}\left(1-\kappa_{c}\right)D_{fvcf}^{f} \end{array} \end{array}$$

$$\begin{array}{c} H_{c}^{\prime f}=H_{cvc}^{\prime f}+H_{cu}^{\prime f}+H_{cvf}^{\prime f}+H_{cvcf}^{\prime f} \end{array}$$

(17) $$\begin{array}{l} \{\begin{array}{l} \;H_{cvc}^{\prime f}=\theta_{c}\left(1-\kappa_{c}\right)D_{cvc}^{f}+\tau_{c}\left(1-\kappa_{c}\right)I_{cvc}^{f}-\gamma_{cH}H_{cvc}^{\prime f}\\ \;H_{cvf}^{\prime f}=\theta_{c}D_{cvf}^{f}+\tau_{c}I_{cvf}^{f}-\gamma_{cH}H_{cvf}^{\prime f}\\ \;H_{cu}^{\prime f}=\theta_{c}D_{cu}^{fc}+\tau_{c}I_{cu}^{f}-\gamma_{cH}H_{cu}^{\prime f}\\ H_{cvcf}^{\prime f}=\theta_{c}\left(1-\kappa_{c}\right)D_{cvcf}^{f}+\tau_{c}\left(1-\kappa_{c}\right)I_{cvcf}^{f}-\gamma_{cH}H_{fvcf}^{\prime f} \end{array} \end{array}$$

Taking


$$\begin{array}{c} I_{c}^{f}=I_{cvc}^{f}+I_{cu}^{f}+I_{cvf}^{f}+I_{cvcf}^{f}\\ D_{c}^{f}=D_{cvc}^{f}+D_{cu}^{f}+D_{cvf}^{f}+D_{cvcf}^{f}\\ H_{c}^{f}=H_{cvc}^{f}+H_{cu}^{f}+H_{cvf}^{f}+H_{cvcf}^{f}\\ I_{f}^{c}=I_{fvc}^{c}+I_{fu}^{c}+I_{fvf}^{c}+I_{fvcf}^{c}\\ H_{f}^{c}=H_{fvc}^{c}+H_{fu}^{c}+H_{fvf}^{c}+H_{fvcf}^{c} \end{array}$$

We get the equation for fully recovered class from both diseases.


(18) $$\begin{array}{l} R^{\prime }=\gamma_{c}I_{c}^{f}+\gamma_{cD}D_{c}^{f}+\gamma_{cH}H_{c}^{f}+\gamma_{f}I_{f}^{c}+\gamma_{fH}H_{f}^{c} \end{array}$$

#### 2.1.2. Dose-specific (COVID-19) vaccine effectiveness

Most existing vaccines are found to be effective against COVID-19 disease; however, emergence and persistent spread of new SARS-CoV-2 variants render these vaccines less effective against the circulating strain (19, 20). The effectiveness of COVID-19 vaccines against infection also varies by the number of doses. Our study considers the case where the circulating strain is Omicron (B.1.1.529) variant. The first dose of COVID-19 has been deemed ineffective against Omicron infection, but the vaccine effectiveness against second, third, and fourth doses increases and will be assumed to be 0.06, 0.39, and 0.49, respectively (21, 22). To be more generic, the COVID-19 vaccine effectiveness in our model is defined as follows:


$$\phi_{c}=\rho_{1}*ef_{1}+\rho_{2}*ef_{2}+\rho_{3}*ef_{3}+\rho_{4}*ef_{4},$$

where *ef*_1_, *ef*_2_, *ef*_3_, and *ef*_4_ represent the effectiveness of doses 1, 2, 3, and 4, respectively, against infection and ρ_1_, ρ_2_, ρ_3_, and ρ_4_ represent vaccine coverage with doses 1, 2, 3, and 4, respectively.

#### 2.1.3. Recovery rate of diagnosed/isolated individuals

As documented in (23), for COVID-19 laboratory-based testing, patient samples must be first transported to the laboratory, and therefore, it takes 1–3 days to receive test results. So, in what follows, we take diagnose rate δ_*c*_=1/2 *day*^−1^ as the baseline assumption. Since we are assuming only flu and COVID-19 are co-circulating, a COVID-19 test negative result implies the flu test positive; hence, we assume the same diagnostic rate for influenza. Furthermore, to calculate the average infectious period 1/γ_*cD*_ of the COVID-19 test positive/isolated cases, we use


$$1/\gamma_{c}=\text{average infectious period for COVID-19}$$

and


$$1/\delta_{c}=\text{average time to test positive of a COVID-19 infection},$$

Therefore,


$$1/\gamma_{cD}=1/\gamma_{c}-1/\delta_{c},\;\gamma_{cD}=\frac{\gamma_{c}\delta_{c}}{\delta_{c}-\gamma_{c}}.$$

As individuals are tested before they recover, 1/γ_*c*_>1/δ_*c*_.

### 2.2. Parameters and initial conditions

We have used some of the data from the province of Ontario, Canada, as a case study. We take the following initial values for vaccinated and unvaccinated state variables. We assume *N*_0_ is the total population of Ontario. From (24), 84.81% of the total population of Ontario will be vaccinated against COVID-19 with dose one, two, three, or four by 19 July 2022. So, initially, a proportion of the population vaccinated against COVID-19 is *V*_*c*_ = 0.8481 × *N*_0_. As a baseline, we assume that 40% (25) of the population who is vaccinated against COVID-19 also gets the influenza vaccine. Thus, this fraction will move to the *V*_*cf*0_ initially vaccinated against both COVID-19 and influenza classes. This gives:


$$V_{c0}=0.8481\times N_{0}-V_{cf0},V_{cf0}=0.4\times 0.8481\times N_{0}.$$

Furthermore, because a large proportion of the population has received COVID-19 vaccine and only a small proportion (15.19%) is left unvaccinated, we assume that 10% of the remaining (COVID-19) unvaccinated population is vaccinated against influenza only. Hence, initially vaccinated against influenza is as follows:


$$V_{f0}=0.1\times \left(N_{0}-0.8481\times N_{0}\right).$$

Thus, unvaccinated class has the initial value:


$$S_{u0}=N_{0}-V_{c0}-V_{cf0}-V_{f0}.$$

The effectiveness of influenza vaccine against hospitalization range about 35–60% by age (26, 27). In our study, we assume a homogeneous population, so in our simulations, we use 50% as a baseline.

Most of the other parameters are taken from the literature. In particular, we numerically estimated the baseline transmission probability β_*c*_ for BA.5 by inverting the formula of reproduction number Ro and following the approach in (28) and using the change in the transmission probability from the ancestral strain to BA.5 in (29, 30). Some of the parameters relevant to influenza, including τ_*f*_, the rate at which influenza infectious cases hospitalized, and δ_*f*_, diagnosis time of symptomatic infected with influenza, are assumed to be the same as COVID-19 for simplifications. On average, the time a person with COVID-19 stays in hospital is 12 days (31), and 10 days for influenza. Furthermore, a fraction *q* of individuals who are isolated immediately on symptoms before testing is chosen at 50% as a baseline. The parameters of the co-circulation model is given in Table 1.

**Table 1:** Parameters definitions and values with references.

**Parameter list**			
**Parameter**	**Definition**	**Value**	**Source**
β_*c*_	Probability of transmission of COVID	0.1351	Estimated for BA.5
β_*f*_	Probability of transmission of Influenza	0.02–0.035	(32)
*C*	Contact rate	11.58	(33)
η	Protective effect against infection by the coronavirus if vaccinated with influenza	0.297	(18)
ϕ_*c*_	COVID-19 vaccine effectiveness against infection	Section 2.1.2	(34)
ϕ_*f*_	Influenza Vaccine effectiveness against infection (reduction in susceptibility)	0.4–0.6	(35, 36)
γ_*c*_	Recovery rate of COVID-19 infectious individuals	1/7	(28)
γ_*cD*_	Recovery rate of COVID-19 diagnosed isolated individuals	1/5	Section 2.1.3
γ_*cH*_	Recovery rate of COVID-19 hospitalized individuals	1/12	(31)
γ_*f*_	Recovery rate of influenza infectious individuals	1/5	(16)
*q*	Fraction of individuals isolated on symptoms before testing	0.5	Assumed
γ_*fH*_	Recovery rate of influenza hospitalized individuals	1/10	Assumed
δ_*c*_	Diagnose rate of symptomatic infected with COVID	1/2	(37)
δ_*f*_	Diagnose rate of symptomatic infected with influenza	1/2	Assumed
1/*w*	Maximum testing capacity per day	5,000	(38)
θ_*c*_	Rate at which COVID-19 confirmed cases hospitalized	0.0305	Calculated from (39)
τ_*c*_	Rate at which COVID-19 infectious cases hospitalized	0.0305	Calculated from (39)
τ_*f*_	Rate at which influenza infectious cases hospitalized	0.0305	Assumed
κ_*c*_	Vaccine effectiveness against hospitalization with COVID	0.8–0.95	(34)
κ_*f*_	Vaccine effectiveness against hospitalization with influenza	0.35–0.5748	(26, 40)

## 3. Simulation results

**Figure 2:**
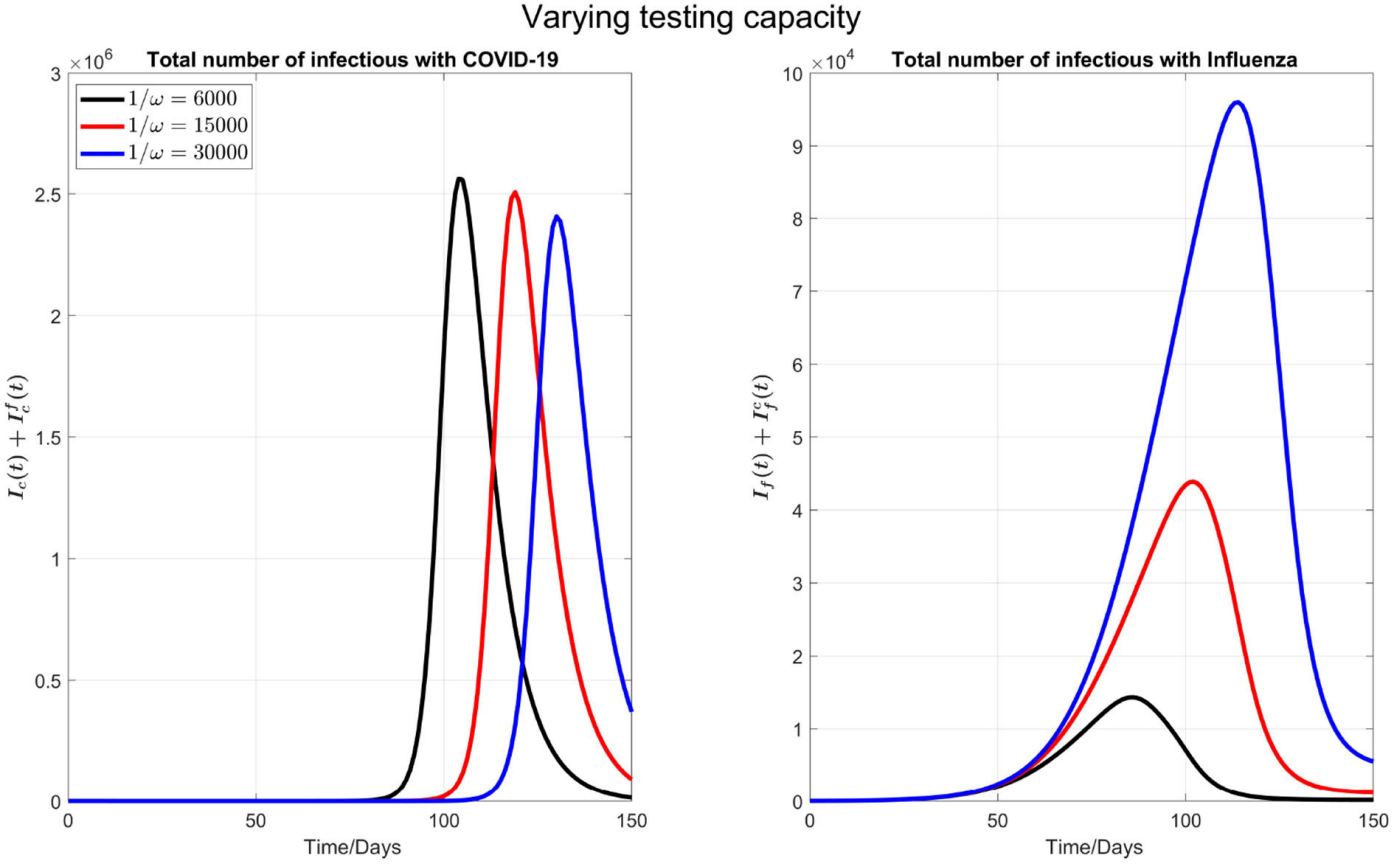
The impact of varying the maximum testing capacity per day on COVID-19 and influenza disease prevalence. Left plot represents the total number of infections with COVID-19 ($I_{c}\left(t\right)+I_{c}^{f}\left(t\right)$) and right plot shows the total number of infections with influenza $\left(I_{f}\left(t\right)+I_{f}^{c}\left(t\right)\right)$.

*Increasing COVID-19 test capacity*: For simulations below, first, we vary the testing capacity from 6,000 to 30,000 in Figure 2.

We observe that by increasing the testing capacity, the peak time of COVID-19 is postponed and the peak value is also reduced (left plot in the Figure 2). On the other side (right plot of the Figure 2), the peak time of influenza cases is delayed but the peak number of influenza infections increases when the COVID-19 test capacity is increased. This is because, when more tests are done per day, more individuals are isolated because the common flu and COVID-19 symptoms are diagnosed earlier, and those who tested negative for COVID-19 will terminate their isolation. So, an early conclusion of COVID-19 negative will increase the force of infection for flu.

**Figure 3:**
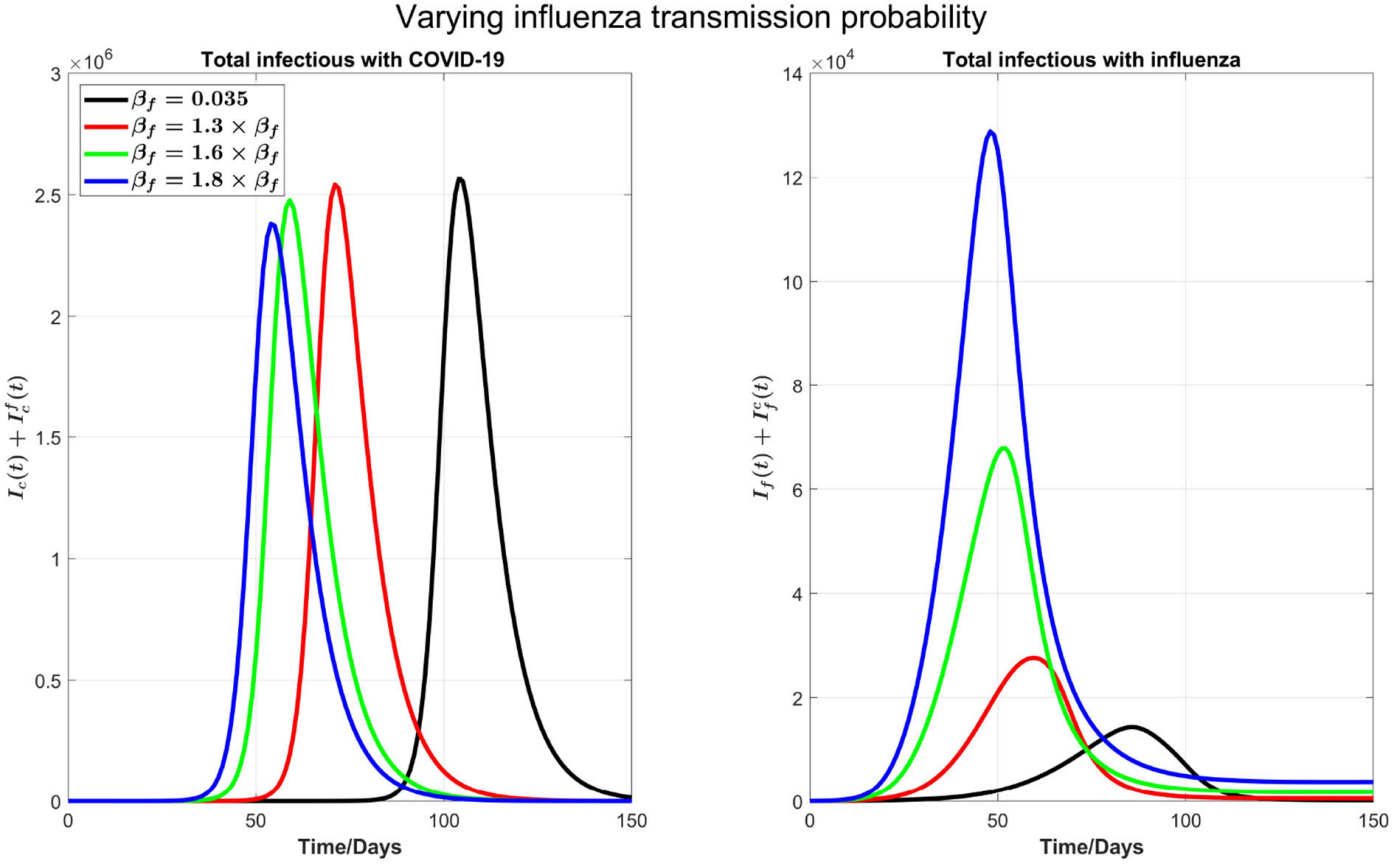
The impact of varying the transmission probability β_*f*_ on COVID-19 and influenza disease prevalence. Left plot represents the total number of infections with COVID-19 ($I_{c}\left(t\right)+I_{c}^{f}\left(t\right)$) and right plot shows the total number of infections with influenza $\left(I_{f}\left(t\right)+I_{f}^{c}\left(t\right)\right)$.

*Varying flu transmission rate*: Next, as shown in Figure 3, we consider varying the transmission probability of influenza β_*f*_. This consideration is motivated by the significantly low flu cases during the COVID-19 pandemic due to social distancing. Consequently, there is a possibility that in the coming flu season, the population has larger than normal susceptibility to the flu. In our simulations, we kept testing capacity 6,000 as a baseline.

**Figure 4:**
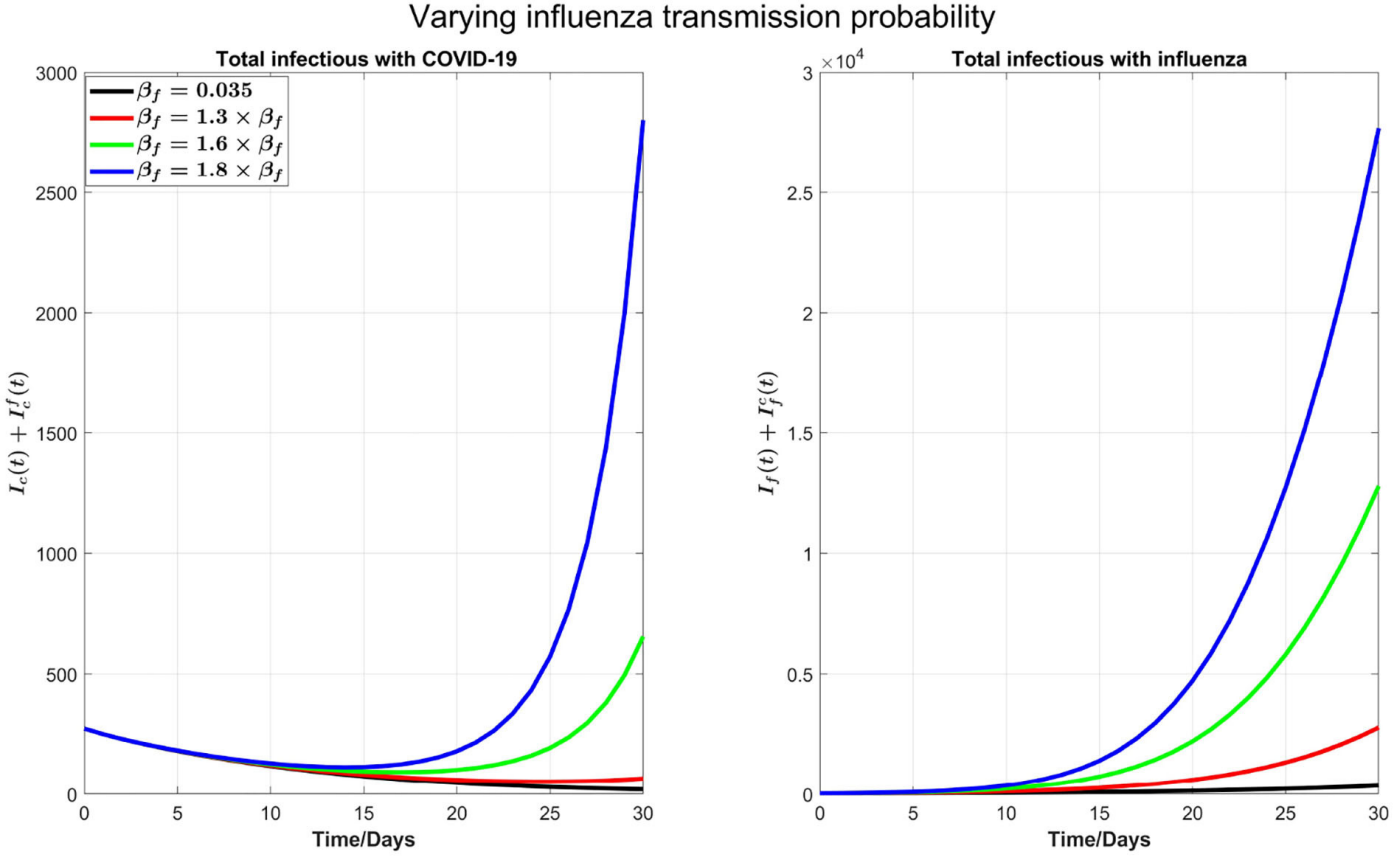
A plot with high resolution for the case in Figure 3 for 30 days. Note that the scale of infections in this plot is some orders of magnitude smaller than in Figure 3.

We can see from the right plot of Figure 4 that influenza cases increases and peak earlier by increasing β_*f*_, which is obvious. However, by increasing transmission probability of influenza, COVID-19 cases (left plot) peak earlier with visible higher peak value. For example, if β_*f*_ is increased by 30%, from 0.035 to 0.0455, then COVID-19 infections peak about 30 days earlier. Again this is attributed by the testing delay due to the high volume of influenza infections: The higher the transmission probability for the flu, the earlier the peak time for flu outbreak and the higher the number of infections with flu-like symptoms to slow down the COVID-19 testing, leading to a delay in full isolation of the patients with COVID-19. Figure 4 shows the impact of increasing β_*f*_ on influenza and COVID-19 infections with high resolution in first 30 days.

### 3.1. The influenza vaccine and COVID-19 booster coverage

We now consider the issue of minimal and optimal coverage of the booster (third) and fourth doses of COVID-19 and influenza vaccines to control both diseases or to reduce the burden of both diseases together in a single season.

**Figure 5:**
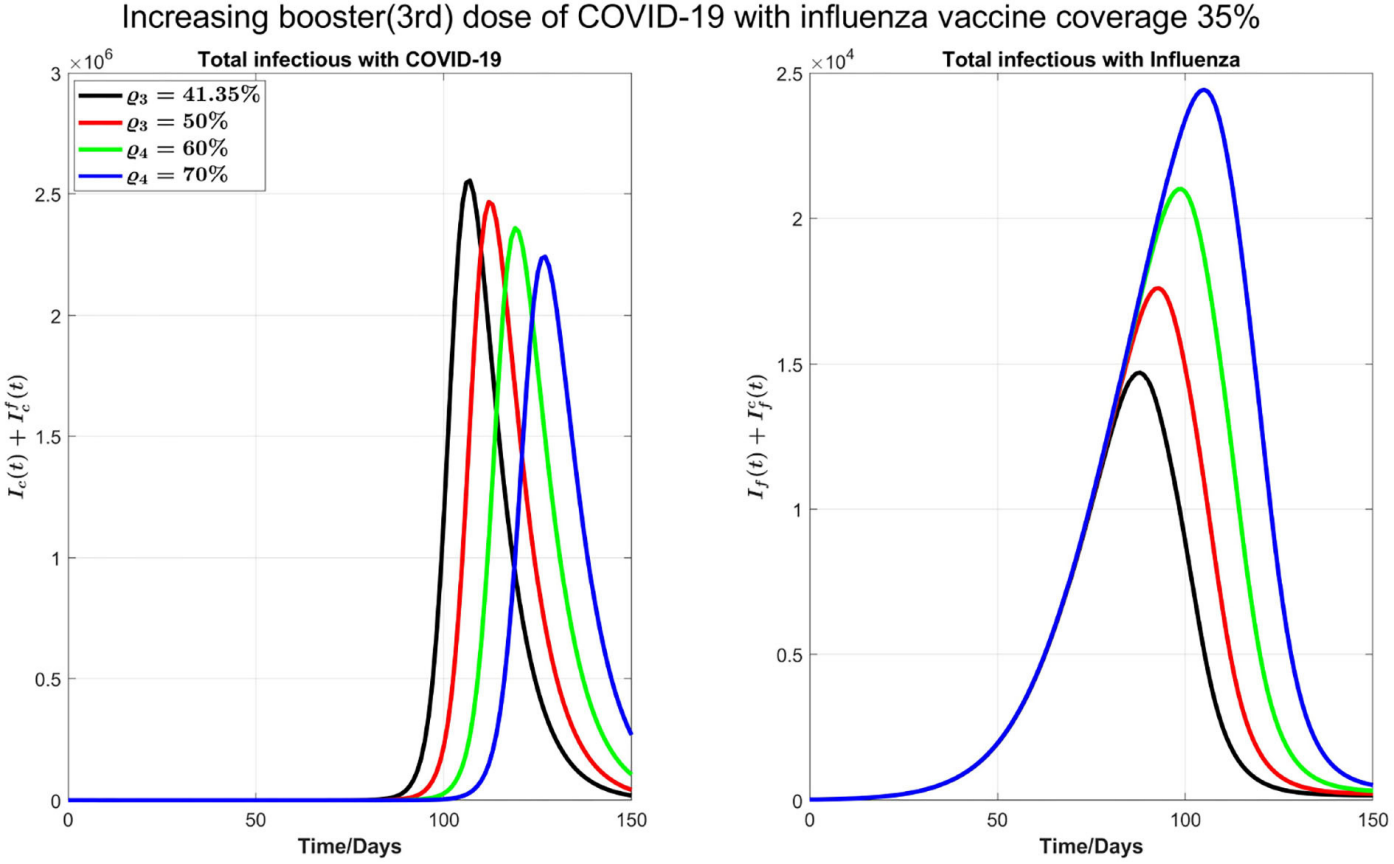
Impact of increasing booster dose when influenza vaccine coverage is 35.43%.

In Figure 5, we start by considering 40% of individuals who are vaccinated against COVID-19 with first, second, or third dose also get the influenza vaccine. As of 19 July 2022, 84.81% of Ontario residents are fully vaccinated against COVID-19. As such, we consider 33.92% of Ontario residents are vaccinated against influenza from this fraction of population, and 10% of the remaining 15.19 % who are not vaccinated against COVID-19 receive influenza vaccine. Thus, the overall population initially vaccinated against influenza is 33.92%+1.519% = 35.43%. Finally, the proportion of the Ontario population receiving booster dose is 41.35%. In our simulations, we will consider this coverage to be increased to 50, 60, and 70%, respectively, to see the impact of increasing booster coverage on the prevalence of both COVID-19 and influenza.

**Figure 6:**
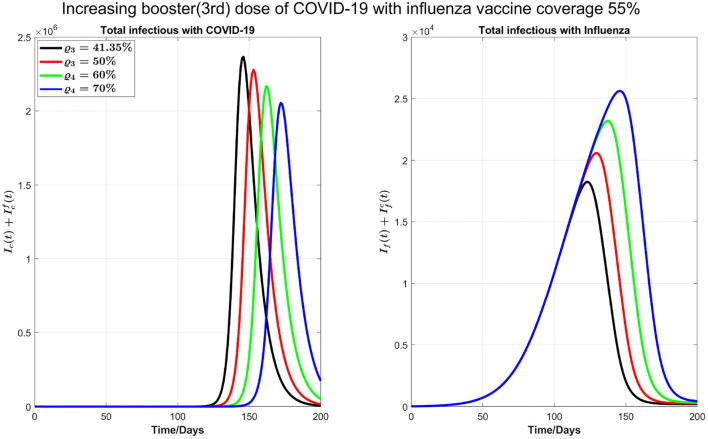
Increasing booster dose when influenza vaccine coverage also increases to 55%.

In the left plot of Figure 6, we observe delaying the COVID-19 peak and reducing the peak size by increasing COVID-19 booster coverage. On the other hand, in the right plot, we observe an increase of influenza peak value with increasing COVID-19 booster coverage; increasing the coverage from 41.35 to 70% will see flu peak value changes from close to 15,000 to 25,000. This is because when we increase the COVID-19 booster coverage, there will be less COVID-19 infections and hence less isolation, so the increase in the population susceptibility to flu.

**Figure 7:**
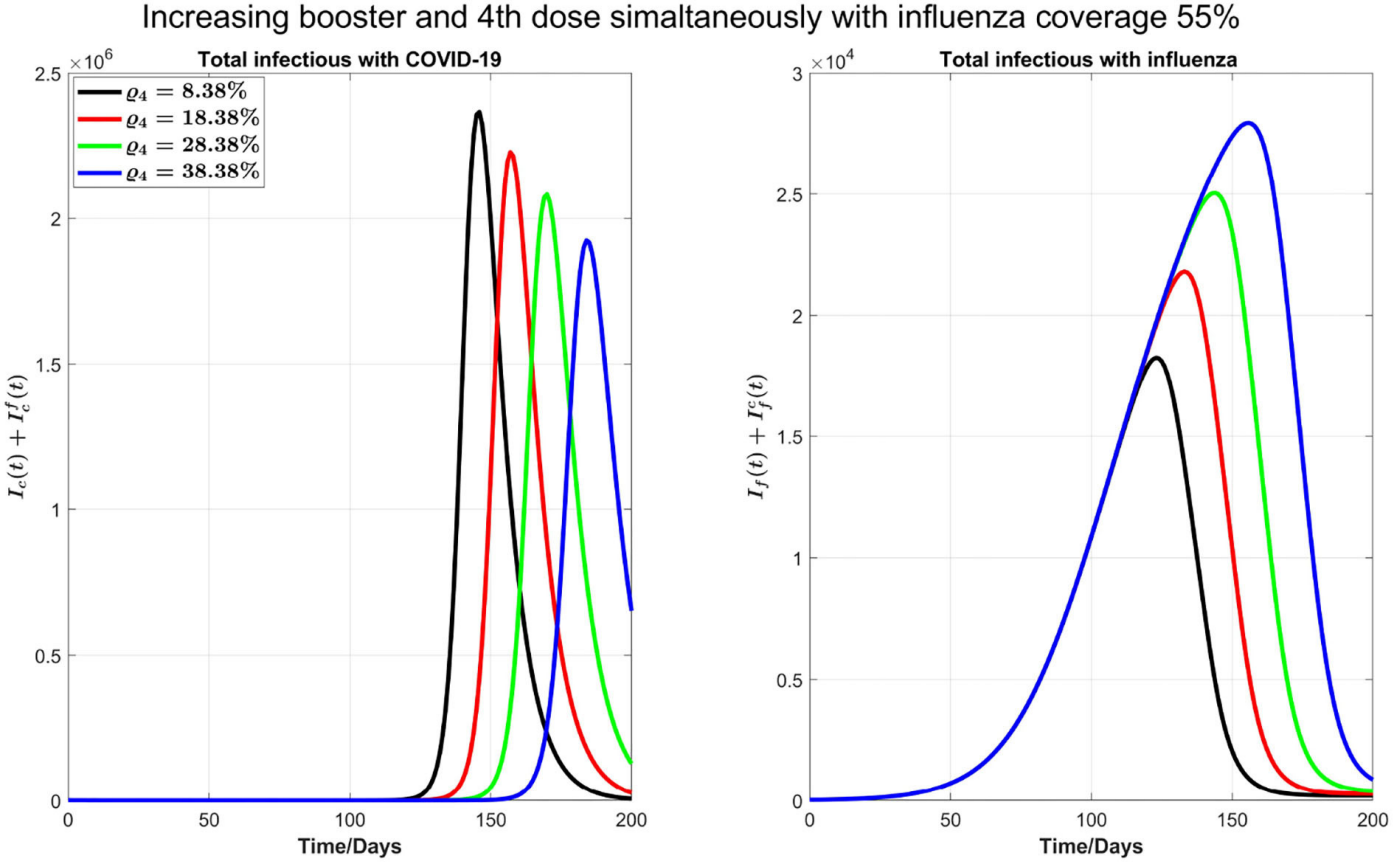
Impact of increasing coverage of COVID-19 booster (third) and fourth doses simultaneously with 55% influenza vaccine coverage on COVID-19 and influenza infections.

Next, as shown in Figure 7, we investigate the impact of increasing the influenza vaccine coverage to 55% from 35%, along with the increase of booster dose of COVID-19. We observe that, though there is no significant impact of increasing influenza vaccine coverage on peak values of both diseases and the peak size of influenza infections increases by increasing influenza vaccine coverage, peak times for both diseases are remarkably delayed. Similar results in Figure 7 are observed by increasing 10% coverage of booster (third dose) and fourth doses of COVID-19 and influenza coverage by 55%.

**Figure 8:**
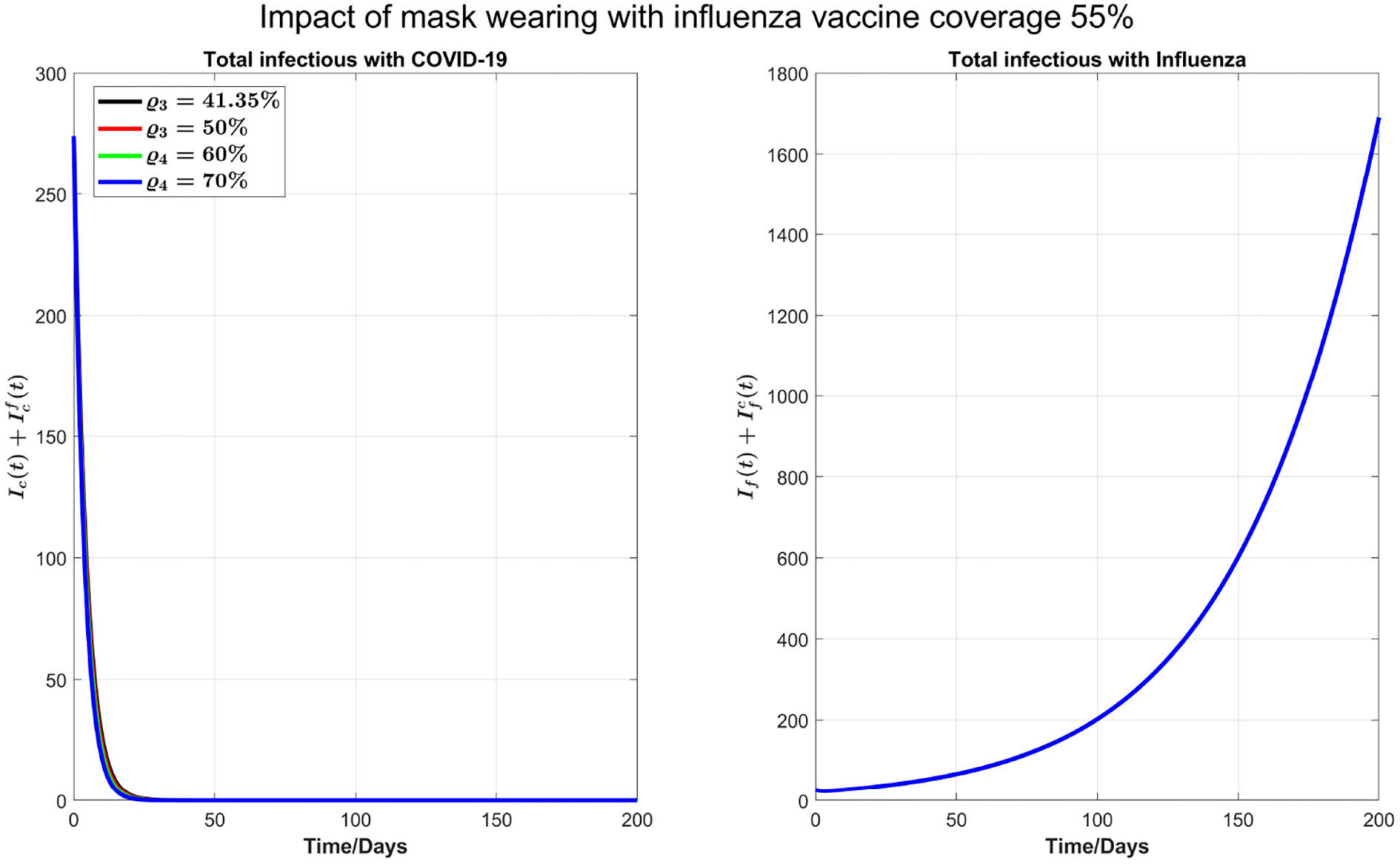
Impact of 20% mask-wearing with an increasing coverage of COVID-19 booster (third dose) and 55% influenza vaccine coverage on COVID-19 and influenza prevalence.

We also carry out some simulations to assess the impact of personal protections such as mask-wearing along with increasing the booster dose of COVID-19 and influenza vaccine coverage by 55%. We note that mask-wearing is no longer compulsory in Ontario; however, mask-wearing is strongly recommended. Reducing β_*f*_ and β_*c*_ by 20%, as shown in Figure 8, there will be no COVID-19 outbreak under a variety of booster coverage, and we also see a noticeable reduction in influenza cases. On the other hand, we observed through simulations result (not shown here) that if influenza coverage is kept at 35%, then there is a high increase in influenza infections. So, we conclude that to control outbreaks of both diseases, an increase in influenza vaccine coverage and continuing the mask-wearing to reduce the COVID-19 and flu transmission by 20% can play a considerable role even without increasing the COVID-19 booster dose or fourth dose.

**Figure 9:**
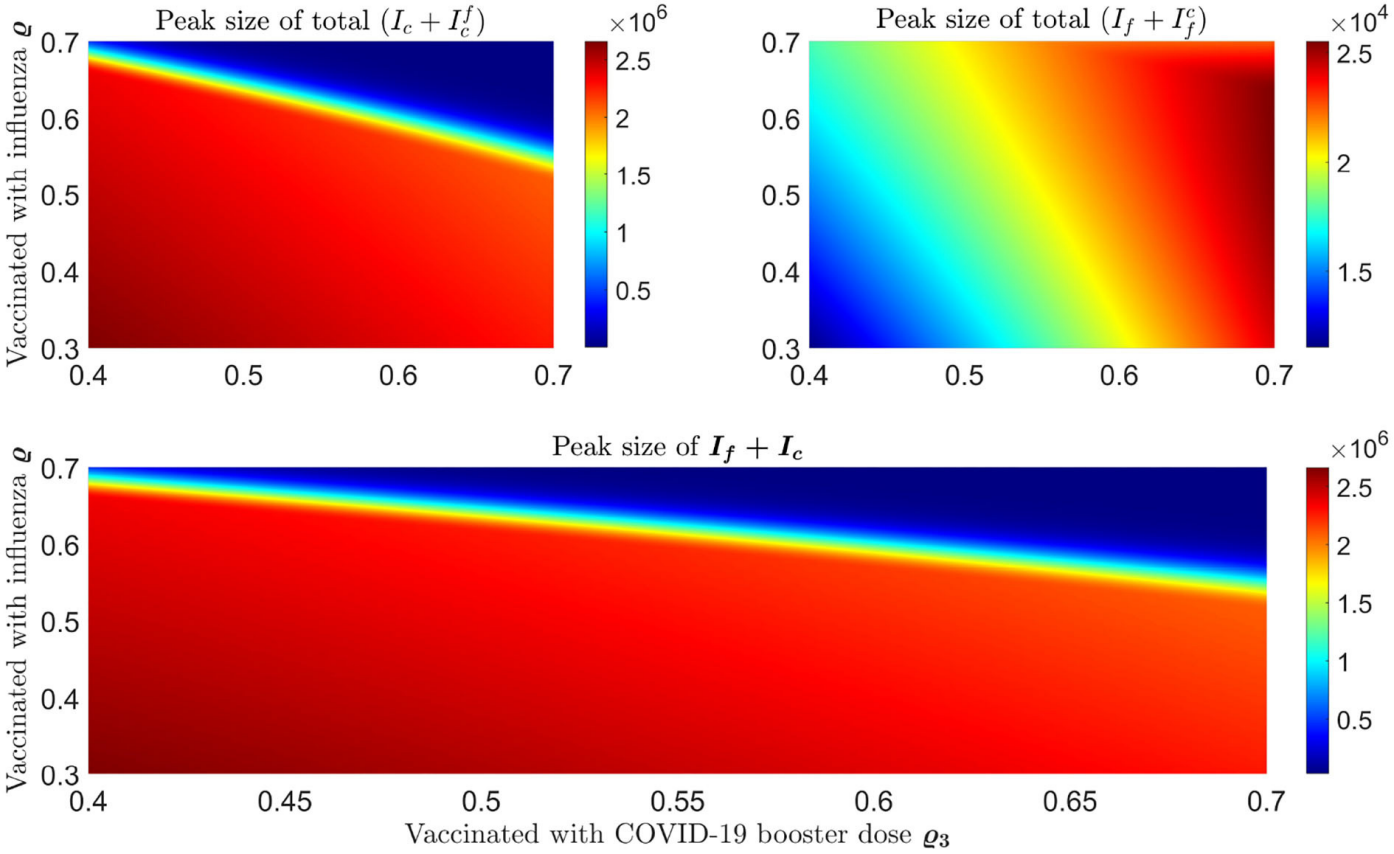
Impact of increasing proportion of individuals vaccinated with the first booster dose of COVID-19 and influenza vaccines on the peak size of infections. The **top left** plot represents the total number of infections with COVID-19 ($I_{c}\left(t\right)+I_{c}^{f}\left(t\right)$) and the **top right** plot shows the total number of infections with influenza $\left(I_{f}\left(t\right)+I_{f}^{c}\left(t\right)\right)$. **Bottom** panel shows the peak size of overall total COVID-19 and influenza cases ($I_{c}\left(t\right)+I_{c}^{f}\left(t\right)+(I_{f}\left(t\right)+I_{f}^{c}\left(t\right)$).

**Figure 10:**
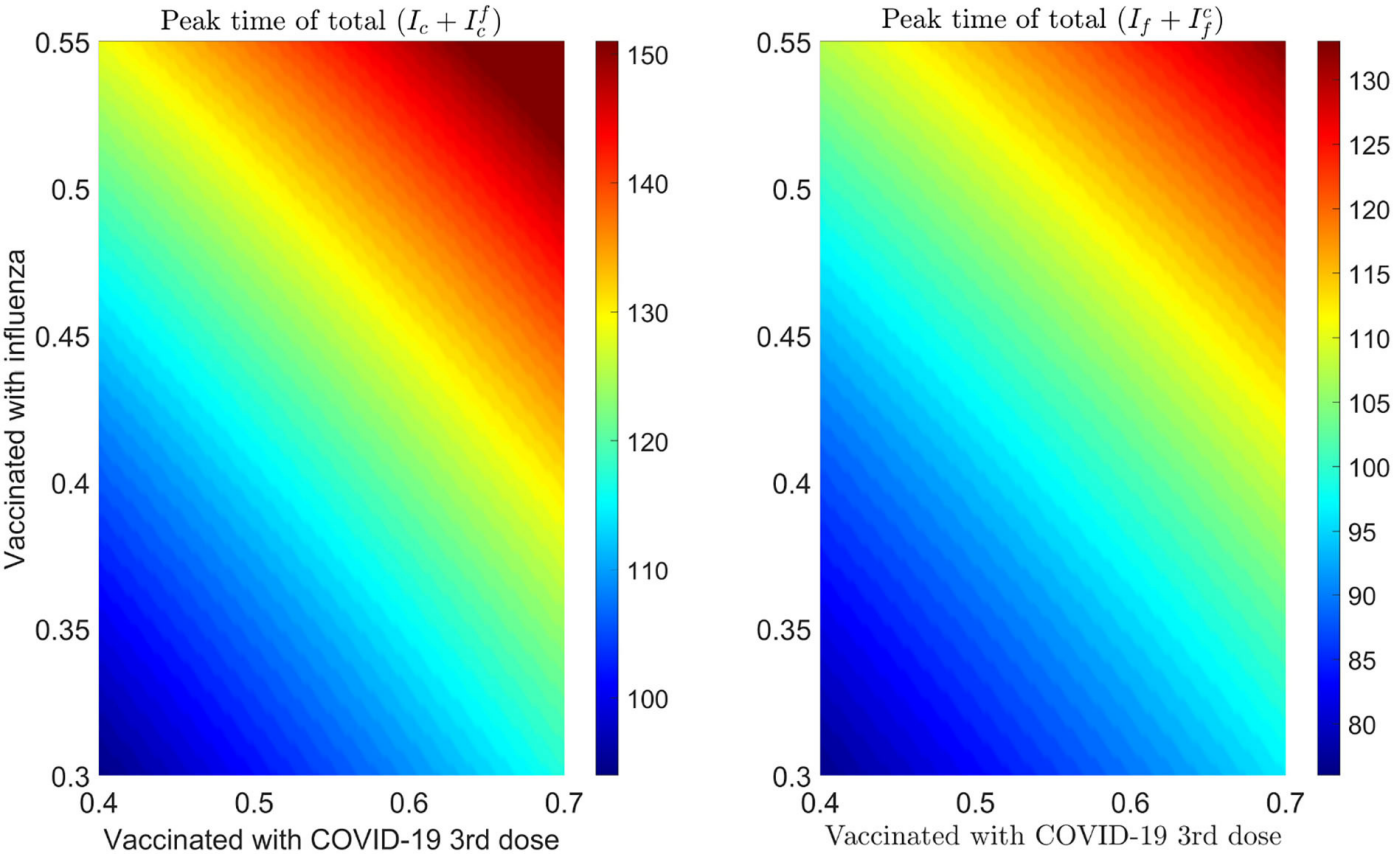
Impact of increasing proportion of individuals vaccinated with the first booster dose of COVID-19 and influenza vaccines on the peak time of infections. **Left** plot represents the total number of infections with COVID-19 ($I_{c}\left(t\right)+I_{c}^{f}\left(t\right)$) and **right** plot shows the total number of infections with influenza $\left(I_{f}\left(t\right)+I_{f}^{c}\left(t\right)\right)$.

We plot some color-coded simulations to visualize the variation of total infections of COVID-19 and influenza at the peak sizes and overall total infections in Figure 9. We vary the proportion of individuals vaccinated with the third dose of COVID-19 vaccine (horizontal axis) with the proportion vaccinated against influenza (vertical axis) and examine the impact on individuals infected with COVID-19 ($I_{c}+I_{c}^{f}$), the upper left panel in Figure 9, and influenza ($I_{f}+I_{f}^{c}$), right panel in Figure 9. The peak size of individuals infected with COVID-19 decreases with an increase in both the proportion of individuals vaccinated with COVID-19 and influenza vaccines. Based on our model results, it is possible to lower the overall peak size and that of individuals infected with COVID-19 when we vaccinate about 70% of the population with the third dose of the COVID-19 vaccine, together with vaccinating about 65% of the population with influenza vaccine (see Figure 9 upper left panel and bottom panel). Alternatively, increasing the proportion of individuals vaccinated with COVID-19 and/or influenza vaccines increase the peak size of individuals infected with influenza (see Figure 9, top right panel). Furthermore, by varying the same proportion of individuals with the booster dose of COVID-19 and influenza vaccine, we plot Figure 10 [the peak time (the time when there would be a maximum number of total COVID-19 infections) (left panel), and total influenza infections (right panel)].

**Figure 11:**
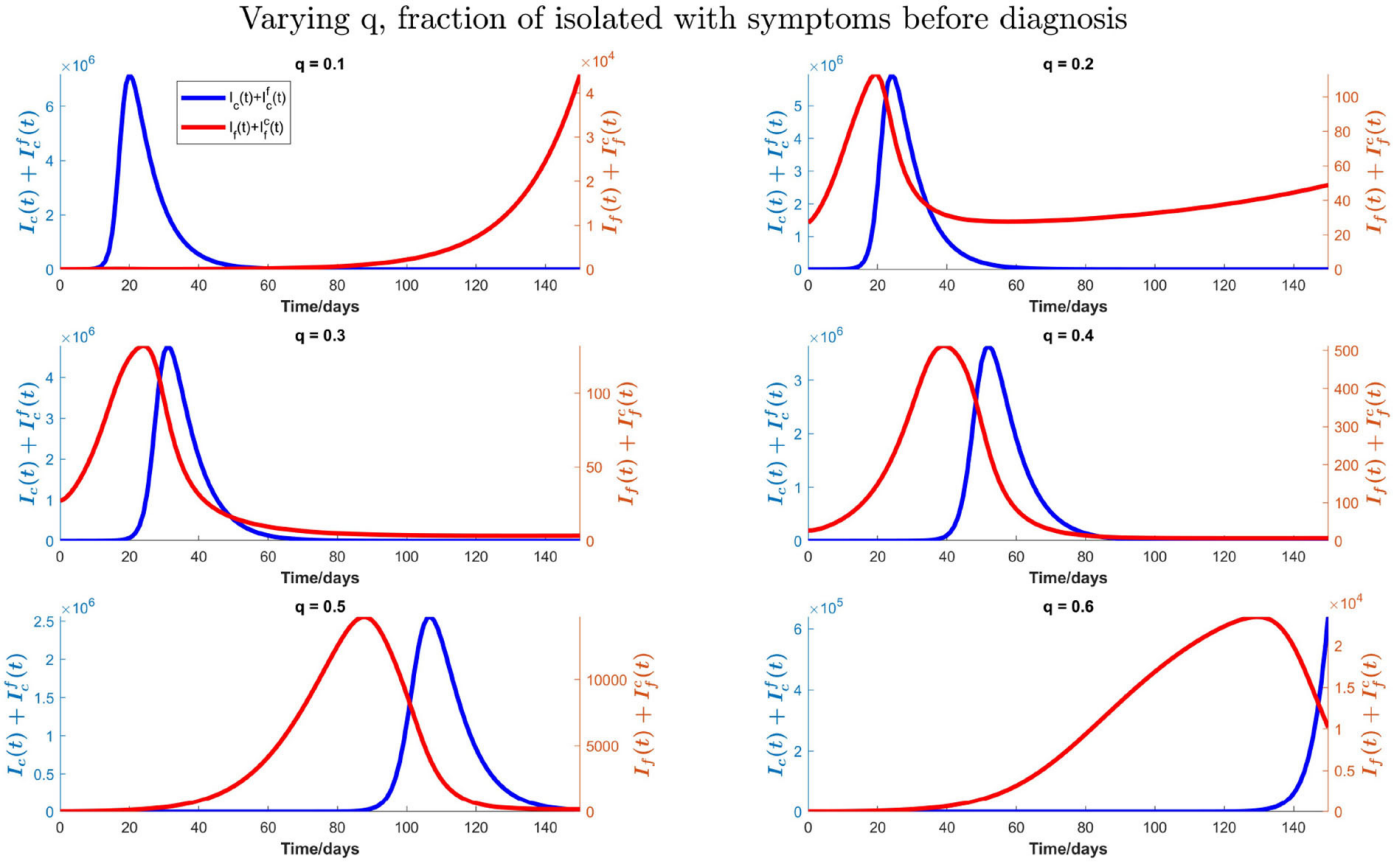
Impact of varying fraction (q) of individuals who isolate them on symptoms of influenza and COVID-19 before testing on total influenza and COVID-19 infections when influenza vaccine coverage is 35%.

We also consider the impact of the fraction of isolation “q” of infected individuals with influenza and COVID-19 symptoms before the diagnosis. We vary this fraction from 10 to 60% to produce Figure 11 with influenza vaccine coverage of 35%. In Figure 11, we can observe that the peak time for COVID-19 is postponed; and peak value is reduced with increasing fraction “q.” The peak time for influenza is also postponed, however, against the intuition, the peak number of infections with influenza for *q* = 20%−30% decreases and then increases again by increasing isolation before testing. This is because of the intensive transmission rate of COVID-19 and dominance at the beginning, with much higher number of initial infections as compared with the influenza. So, after getting recovered from COVID-19, there would be more susceptibility to influenza, and influenza infections start to increase and COVID-19 infections start to decrease. With *q* = 40%−60%, there is a substantial delay in the COVID-19 peak. So, more infections with influenza can be seen earlier because of more susceptibility to influenza. Although there is also a delay in influenza peak with increasing “q,” similar results (not shown here) are found with influenza vaccine coverage of 55%.

### 3.2. Sensitivity analysis

**Figure 12:**
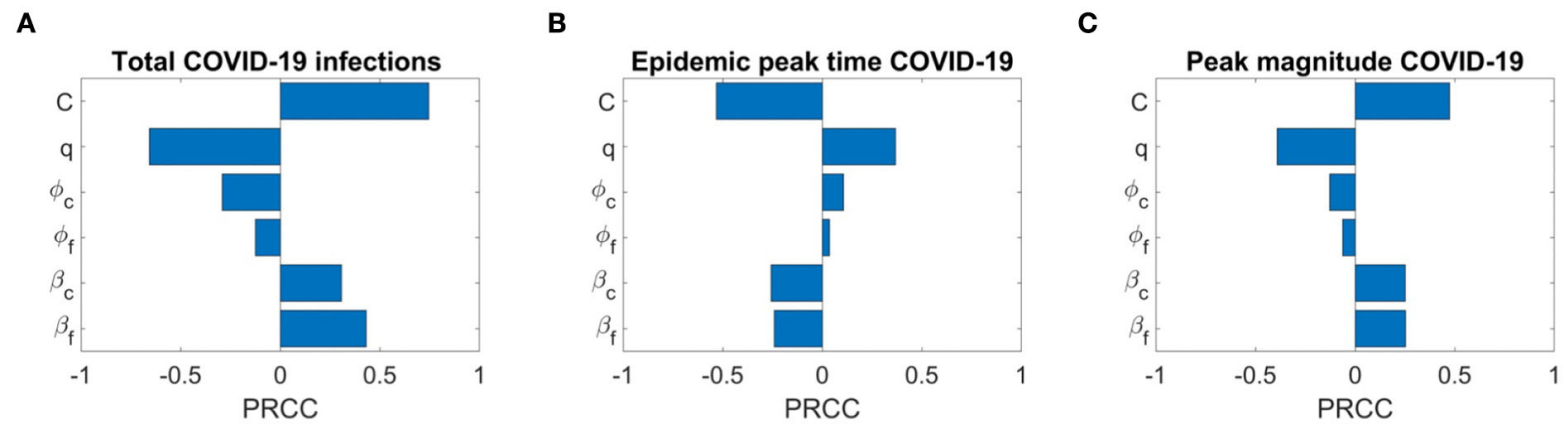
Sensitivity analysis of three epidemic outcomes of COVID-19. **(A–C)** Total COVID-19 infections, the epidemic peak time of COVID-19, and the peak magnitude of COVID-19 respectively. The sensitivity analysis is done with 2,000 bins.

**Figure 13:**
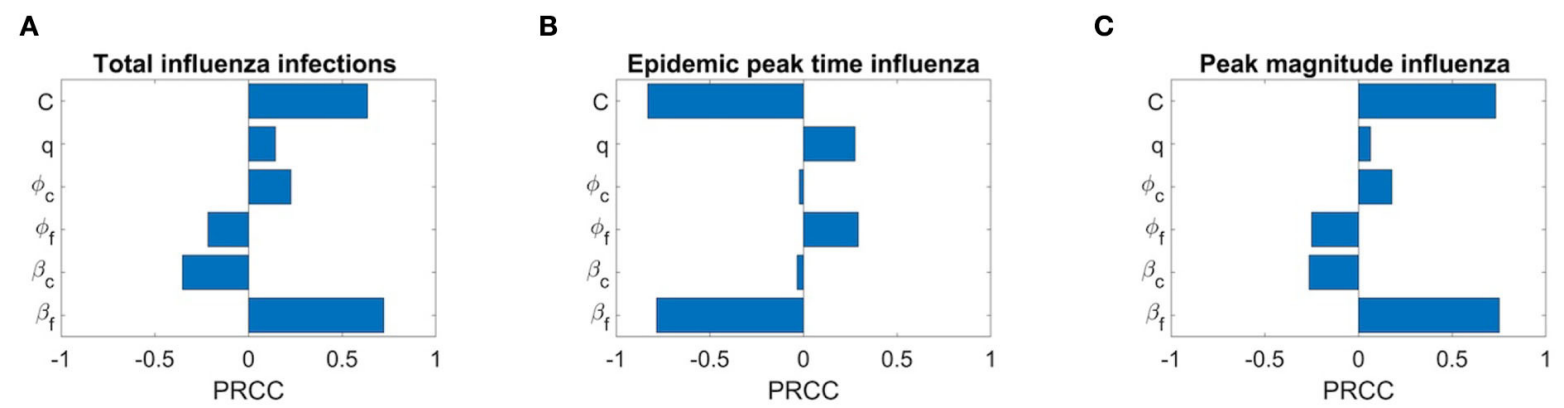
Sensitivity analysis of three epidemic outcomes of influenza. **(A–C)** Total number of influenza infections, epidemic peak time of influenza, and peak magnitude of influenza, respectively.

We also conduct the sensitivity analysis using Latin Hypercube Sampling (LHS) and partial rank correlation coefficients (PRCC) method on different important epidemic outcomes to public health, like the total number of infections, peak number of infections (peak magnitude), and peak time to identify the key parameters of epidemic with the hope of determining public health measures that can be implemented to control or eliminate the outbreaks of these diseases. PRCC is an efficient sensitivity analysis method based on sampling, which assigns a value between −1 and +1 for each parameter. A positive PRCC value indicates a positive correlation of the parameter with disease maintenance, whereas a negative value indicates a negative correlation with the disease. The parameters studied are as follows: β_*f*_, β_*c*_, ϕ_*f*_, ϕ_*c*_, q, and C. Figures 12, 13 show the PRCC indices of these selected parameters on total number of infections (Figure 12A), peak time (Figure 12B), and peak number of infections (Figure 12C) of COVID-19 and influenza, respectively.

Figure 13A determines that the disease transmission probabilities, β_*f*_ and β_*c*_, and contact rate *C* have a positive impacts, while vaccine effectiveness ϕ_*f*_, ϕ_*c*_, and *q* have negative impacts. A significant positive correlation among COVID-19 infections and contact rate *C* and β_*f*_, and a negative correlation with COVID-19 vaccine effectiveness ϕ_*c*_ are evident. A positive correlation with β_*f*_ means more susceptibility to influenza in the population leading to more infections with influenza would also lead to more infections with COVID-19 because of common symptoms and delay in diagnosis. On the other hand, a strong negative correlation of q means as many individuals on symptoms immediately isolate themselves before diagnosis, there would be fewer infections with COVID-19. We can also see that COVID-19 infections are less sensitive to influenza vaccine but increase in influenza coverage would reduce COVID-19 infections. This is because influenza vaccines are assumed to provide some protection against COVID-19 infection too. Figure 13B shows the effects of the PRCC indices on the peak time. The effects of these parameters are reversed, but their relative levels of influence show a similar pattern. Finally, Figure 13C shows the effects of the PRCC indices on the epidemic peak size. The impacts of these parameters closely resemble to those observed in Figure 13C. Next, Figure 13A shows the sensitivity of the chosen parameters to total influenza infections. Here, a strong positive correlation of β_*f*_ and contact rate *C* and a significant negative correlation of ϕ_*f*_ and β_*c*_ with total influenza infections are understandable. But a positive impact of ϕ_*c*_ on influenza infections means that if there are more COVID-19 booster or fourth doses administrated (considering booster and fourth doses because first dose has no and second dose has very low, 0.06%, effectiveness against COVID-19 infection), then it will result in more infections with influenza. Furthermore, here q has a positive correlation with influenza infections. Similarly, sensitivity analysis results for influenza epidemic peak magnitude (Figure 13C) and a reverse pattern for influenza peak time (Figure 13B) are obtained.

## 4. Discussion

In the present study, we developed a deterministic mathematical model to describe the dynamics of COVID-19 and influenza transmission when both are present in the same season, and a certain percentage of the population has received the influenza, COVID-19, or both vaccines.

Worldwide, the influenza virus, through seasonal waves of infection, generates a significant toll of cases and deaths (41). According to the World Health Organization, before the COVID-19 pandemic, there were an estimated 1 billion cases yearly, of which 3–5 million resulted in 290,000–650,000 influenza-related respiratory deaths (42). In the Northern and Southern Hemispheres, the flu season usually runs from October to April and April to September, respectively.

COVID-19 has perturbed seasonal influenza activity (43): in 2020 and 2021, this remained low historically at international levels (44–49). These changes were attributed to the widespread implementation of NPIs such as physical distancing, masking requirements, and lockdowns to mitigate the transmission of SARS-CoV-2. More recently, however, and coinciding with relaxation in public health mitigation, a substantial simultaneous burden of influenza and SARS-CoV-2 has been observed in the Southern Hemisphere. The June 2022 Australian Influenza Surveillance Report stated that from mid-April 2022, the weekly number of laboratory-confirmed influenza cases in Australia had occurred earlier than usual and exceeded the 5-year average (50). In the Northern Hemisphere, in Canada, after lifting public health measures at the beginning of March 2022, influenza virus circulation increased, reaching the seasonal epidemic threshold in April with an unusual peak in May 2022 (51).

Although influenza and COVID-19 viruses belong to different families and have some differences, with SARS-CoV-2 having a much higher basic reproduction number, longer incubation period, and shorter interval between symptoms onset and infectivity (52), COVID-19 shares many significant clinical and epidemiological features with influenza, such as transmission routes and symptoms. Clinical differentiation of these two respiratory diseases can be a challenge, particularly in the early stage, based on a common diagnosis in the absence of laboratory evidence and isolation of a specific pathogen. Therefore, a laboratory diagnostic test may be required to rule out the suspicion and establish a definitive diagnosis. Some sophisticated mathematical/statistical techniques, such as machine learning-based decision modeling approaches, have been able to distinguish between influenza and COVID-19 cases (53, 54).

In terms of clinical public and global health, even though available vaccines do not provide complete protection against infection, they can reduce the severity of the disease significantly. As influenza virus also mutates frequently, an updated flu vaccine is required every year, considering which strains are anticipated to circulate in the community. Thus, the effectiveness of the flu vaccine varies depending on how well the vaccine matches with circulating strains or who is being vaccinated (age or health characteristics of the vaccinated individual) (55). According to health professionals, receiving influenza and COVID-19 vaccines is a cornerstone to protect against illness of both diseases and potentially their severe consequences, including hospitalization and fatality (56, 57).

Therefore, reducing the burden of COVID-19 and influenza will depend primarily on increasing vaccination coverage. A mathematical model (58) has modeled the interaction between SARS-CoV-2 and influenza during the early phase of the COVID-19 pandemic. Using a population-based modeling approach, the authors found a 2- to 2.5-fold population-level increase in COVID-19 transmission associated with influenza co-circulation, warranting the importance of being immunized against influenza. Influenza vaccination can represent, indeed, an important public health intervention to, at least partially, relieve and mitigate against the burden generated by SARS-CoV-2 and influenza co-circulation (14). As found by a systematic review of the literature, influenza immunization can also, indeed, confer protection against COVID-19 (59).

In our model, we took into account the multi-factorial interactions between influenza and COVID-19 in terms of additional epidemiological, clinical, and organizational burdens due to their comparable early symptoms, which can present a clinical challenge in determining the patient's disease type and overwhelm testing capacity, lowering the diagnosis rate. In this scenario, we observe that if testing capacity is increased per day, it will delay the peak of COVID-19 in addition to reducing it. On the other hand, because of early detection and lack of isolation, influenza cases can grow. Agreeing with (60), the low levels of influenza activity in the previous 2 years of the COVID-19 pandemic may result in an increased proportion of susceptible individuals. Our simulations with increasing influenza transmissibility show that this will bring the peaks of influenza and COVID-19 earlier, slightly decrease the COVID-19 peak size, but significantly increase the flu peak. Here, we can see the real influence of co-circulation and interaction of COVID-19 and influenza on each other. It is because of the testing delay brought on by the high number of influenza cases. The earlier the flu season's peak and greater the number of infections with flu-like symptoms, the greater the risk of flu transmission, which slows down COVID-19 testing, resulting in the delay of complete isolation of patients with COVID-19, who have not been isolated before the clinical presentation of symptoms and have been continuing their normal daily activities. Furthermore, our simulations stress the importance of vaccine uptake for preventing infection, severe illness, and hospitalization at the individual level and for disease outbreak control at the population level to avoid putting strain on already weak and overwhelmed healthcare systems. As such, ensuring optimal vaccine coverage for COVID-19 and influenza to reduce the burden of these infections is paramount. We showed that by keeping the influenza vaccine coverage about 35% and increasing the coverage of booster or fourth dose of COVID-19 not only reduces the infections with COVID-19 but also can delay its peak time. If the influenza vaccine coverage is increased to 55%, unexpectedly, it increases the peak size of influenza infections slightly, while it reduces the peak size of COVID-19 as well as significantly delays the peaks of both of these diseases. Also, we have shown that personal protection decisions like mask-wearing can mitigate the COVID-19 outbreak and can avert an outbreak of seasonal influenza significantly. In conclusion, an increase in vaccine uptake of both diseases, particularly influenza, can significantly delay the peak time of both COVID-19 and influenza. Mask-wearing coupled with a moderate increase in the vaccine uptake may mitigate COVID-19 and prevent an influenza outbreak.

However, there are some limitations in the present study. To keep this model parsimonious, we did not include latent or asymptomatic stages of infection. We believe including these stages would not overly affect our predictions due to the majority of COVID-19 and flu cases being symptomatic, and therefore requiring diagnosis (61). Future studies may be extended to add these states if needed. We did not consider outflow from the isolation class (before testing) to the recovered class because of the long delay in diagnosis. Although we did some simulations by incorporating it into our model (not included in the article), there is not much difference between these and our key findings of the given model in this study, except we see more delay in peak times of both diseases. And by increasing influenza vaccine coverage from 35 to 55%, there is a delay in influenza cases instead of a slight increase in influenza peak as in our original model. Also, with 55% influenza vaccine and 20% masking, there would be no outbreak of COVID-19 and influenza even if booster and fourth doses coverage are kept moderate.

Our study has important practical implications for public health policy: It shows that effectively managing and controlling both influenza and COVID-19 outbreaks during the same season depend on ensuring optimal strategies in terms of vaccine coverage.

## Data availability statement

The original contributions presented in the study are included in the article/supplementary material, further inquiries can be directed to the corresponding author.

## Author contributions

BM, WW, and JW originated the research idea. The model formulation was carried out by BM. Numerical simulations were performed by BM with the help of JD and WW. The writing of the manuscript was completed by BM, reviewed and edited by JW, NB, and WW. The literature review was done by BM and NB. The project was supervised by JW, and the project was led by WW. All authors provided critical feedback and helped shape the research and analysis. All authors gave final approval for publication and agreed to be held accountable for the work performed therein.
